# A novel small molecule RAD51 inactivator overcomes imatinib-resistance in chronic myeloid leukaemia

**DOI:** 10.1002/emmm.201201760

**Published:** 2013-01-22

**Authors:** Jiewen Zhu, Longen Zhou, Guikai Wu, Heiko Konig, Xiaoqin Lin, Guideng Li, Xiao-Long Qiu, Chi-Fen Chen, Chun-Mei Hu, Erin Goldblatt, Ravi Bhatia, A Richard Chamberlin, Phang-Lang Chen, Wen-Hwa Lee

**Affiliations:** 1 Department of Biological Chemistry, School of Medicine, University of CaliforniaIrvine, CA, USA; 2 Division of Hematology and Bone Marrow Transplantation, City of Hope National Medical CenterDuarte, CA, USA; 3 Department of Chemistry, University of CaliforniaIrvine, CA, USA

**Keywords:** cancer, CML, inhibitor, RAD51, small molecule

## Abstract

RAD51 recombinase activity plays a critical role for cancer cell proliferation and survival, and often contributes to drug-resistance. Abnormally elevated RAD51 function and hyperactive homologous recombination (HR) rates have been found in a panel of cancers, including breast cancer and chronic myeloid leukaemia (CML). Directly targeting RAD51 and attenuating the deregulated RAD51 activity has therefore been proposed as an alternative and supplementary strategy for cancer treatment. Here we show that a newly identified small molecule, IBR2, disrupts RAD51 multimerization, accelerates proteasome-mediated RAD51 protein degradation, reduces ionizing radiation-induced RAD51 foci formation, impairs HR, inhibits cancer cell growth and induces apoptosis. In a murine imatinib-resistant CML model bearing the T315I Bcr-abl mutation, IBR2, but not imatinib, significantly prolonged animal survival. Moreover, IBR2 effectively inhibits the proliferation of CD34^+^ progenitor cells from CML patients resistant to known BCR-ABL inhibitors. Therefore, small molecule inhibitors of RAD51 may suggest a novel class of broad-spectrum therapeutics for difficult-to-treat cancers.

## INTRODUCTION

Homologous recombination (HR) is a critical cellular process, allowing cells to cope with genotoxic stresses by repairing DNA double-stranded breaks (DSBs), interstrand crosslinks, and abnormal replication forks with high fidelity. The biochemical processes of the HR are mediated by a repertoire of conserved factors including the essential recombinase RAD51, an ATPase with DNA binding activity. RAD51 can multimerize and form nucleo-filaments on ssDNA, a step critical for the pairing of homologous DNA sequences and the subsequent strand exchange (Baumann et al, 1996; Conway et al, 2004; Haaf et al, 1995; Sung & Robberson, 1995). These RAD51-dependent processes are facilitated by many accessory factors including the breast cancer susceptibility gene product BRCA2. Even though cells deficient in the accessory factors may be viable, those without RAD51 are not, primarily because of the indispensable role of RAD51 in the HR pathway.

Cancers are often under a high level of DNA damage and DNA replication stress, which require functional DNA repair pathways for optimal survival (Luo et al, 2009). Elevated RAD51 expression and enhanced HR rates have been observed in many types of cancers, including chronic myelogenous leukemia (CML), in correlation to high proliferation rate and radio- and chemo-resistance (Klein, 2008; Raderschall et al, 2002; Skorski, 2002; Slupianek et al, 2001; Vispe et al, 1998; Xia et al, 1997). In contrast, normal cells usually do not have such a persistent high level of genotoxic stresses. When normal cells were subjected to genotoxic treatment, DNA damage signals would halt proliferation and induce senescence or apoptosis, partly due to the well-regulated cell cycle control. Hence, interfering with the capability of cancer cells to cope with genotoxic stresses has been proposed for potential cancer treatment. For example, cancers with deficient HR activity are found to be hypersensitive to inhibitors targeting the base excision repair pathway (Bryant et al, 2005). Alternatively, cells may use HR as a critical backup pathway to handle oxidative stresses in the absence of base excision repair activity (Letavayova et al, 2006). Thus, RAD51 is an attractive target molecule for developing tumour-selective inhibitors. Consistent with this idea, prior studies have shown that depletion of RAD51, using small interfering or antisense RNAs, sensitizes cancer cells to IR and promotes cell death (Ito et al, 2005; Sak et al, 2005).

Here we describe the development of a RAD51 specific small molecule inhibitor for potential cancer treatment. RAD51 directly binds to the six conserved BRC repeats of BRCA2 (Chen et al, 1998, 1999). Structural studies of the RAD51-BRC peptide complex demonstrates that the contact motifs at the BRC/RAD51 binding interface mimic those involved in RAD51 multimerization (Conway et al, 2004; Pellegrini et al, 2002; Shin et al, 2003). Indeed, excess BRC peptides can inactivate RAD51, leading to the failure of RAD51 to form nucleo-protein filaments (Davies et al, 2001) and IR-induced foci (Chen et al, 1999). These unique biochemical properties inspired us to design a new strategy to search for small molecule RAD51 inactivators.

## RESULTS

### Identification of a small molecule (IBR2) that binds to RAD51

**Figure 1:**
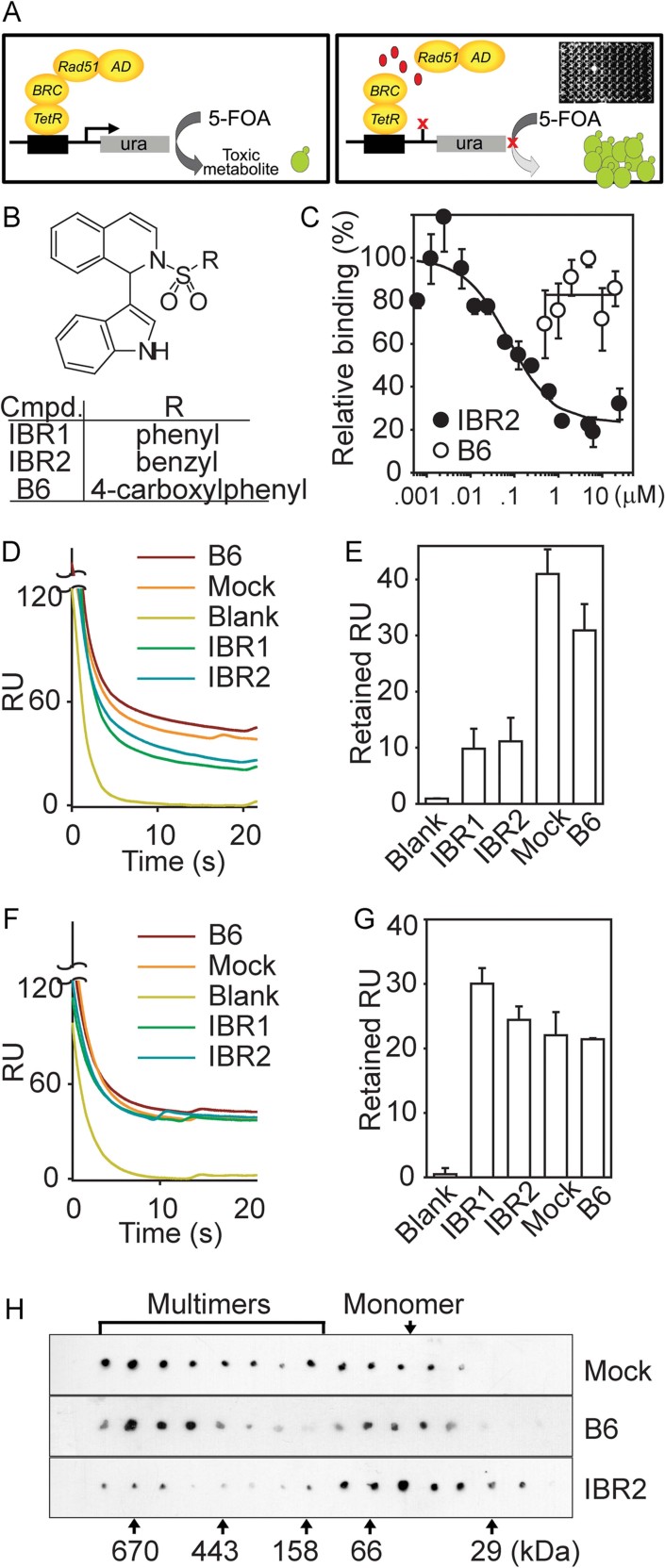
IBR2 binds RAD51 and disrupts RAD51 multimerization **A.** Small molecule RAD51 binders were identified using an inducible reverse yeast two-hybrid system. Compounds that potentially disrupt the BRC-RAD51 interaction were identified by measuring yeast viability. Inset: An example of a positive hit from the screening.**B.** Chemical structures of IBR1/2 and inactive analogue B6.**C.** IBR2, but not B6, competitively disrupts the interaction between His-RAD51 and GST-BRC, with a median competitive inhibition concentration of 0.11 µM.**D–G.** SPR sequential binding assays. Either His-RAD51 or GST-BRC immobilized sensor chips are first mock-pretreated (Mock), or pre-treated with IBR1/2 or B6 (1 µM), and then treated by a single injection of the second protein: GST-BRC or His-RAD51, respectively, or BSA (Blank). SPR signals are expressed in resonance units (RU). Representative SPR dissociation curves are shown in (**D**) and (**F**); and results from two independent experiments are summarized in (**E**) and (**G**). (**D**) and (**E**) Pre-treatment of a RAD51-immobilized chip with IBR1 or IBR2, but not with B6, abolishes the binding of BRC repeat. (**F**) and (**G**) Pretreatment of a BRC-immobilized chip with IBR1, IBR2 or B6 does not affect the binding of RAD51.**H.** IBR2 but not B6 inhibits RAD51 multimerization.

Using a forward chemical-genetics approach, we employed an inducible reverse yeast two-hybrid system, which allows yeast growth when the candidate compound abolishes the interaction between the BRC and RAD51-derived probes (TetR-BRC and a *GAL1-*inducible AD-RAD51 fusion, Fig 1A). When the interaction is not disrupted, the two probes assume normal binding, resulting in transcriptional activation of the TetOp-driven *URA3* gene and subsequent generation of a toxic metabolite via the hydrolysis of 5-fluoroorotic acid (5-FOA; Boeke et al, 1987). Thus, from a chemical library of 24,000 structurally diversified small compounds, we identified two compounds that promoted yeast-growth, IBR1 and IBR2, which share a phenylsulfonyl indolyl isoquinoline structure (Fig 1B and Supporting Information Fig S1). IBR2 was slightly more potent than IBR1 in inhibiting cancer cell growth, and was further studied herein. An IBR analogue (B6), with a carboxyl group at the *para*-position of the phenyl ring (Fig 1B), was identified as an inactive compound and used as a negative control.

To validate that IBR2 disrupts the BRC/RAD51 interaction, we performed a surface plasmon resonance (SPR) competitive binding assay (Vassilev et al, 2004) and compared the RAD51 binding capacity of IBR2 or B6 in competition with BRC repeats (GST-BRC) by injecting a mixture of the small compounds and the GST-BRC protein over a RAD51-modified sensor surface. As shown in Fig 1C, IBR2 effectively competed with the BRC repeats for RAD51 binding with a median competitive inhibition concentration of approximately 0.11 µM, while that of B6 was well above 20 µM. Hence, a single structural modification from a benzyl group (as in IBR2) into a *p*-carboxylphenyl group (as in B6) sufficiently abolished the ability of IBR2 to compete with BRC repeats in RAD51 binding (Fig 1C).

To dissect whether RAD51 or the BRC repeat serves as the actual target of IBR1/2, a sequential SPR binding assay was used to detect the inhibition of protein–protein interaction. In principle, one of the proteins was bound to a sensor chip, and the compounds were allowed to pass over the surface. The second protein was then passed over the surface and the binding was measured. If the compounds bind the immobilized protein, the binding of the second protein will be reduced; otherwise, the binding will be largely retained. Upon pre-treatment with IBR1/2, immobilized RAD51 lost the ability to bind BRC repeat (Fig 1D and E), whereas neither compound affected the binding of RAD51 to immobilized BRC repeat (Fig 1F and G). In contrast, pre-treatment of the inactive compound B6 did not interfere with BRC-RAD51 binding on either chip (Fig 1D–G). These results indicate that IBR1/2 target RAD51 rather than the BRC repeat.

BRC repeats have a highly conserved hairpin structure (*e.g.* 1524-FHTASGK-1530 in BRC4), which is critical for binding to the RAD51 core domain through a hydrophobic pocket formed between β-strand B3 and α-helix A4 of RAD51 (Pellegrini et al, 2002). This domain is also critical for RAD51 multimerization. Since IBR2 competitively inhibits the binding of BRC to RAD51, we reasoned that the compound may mimic and compete with BRC repeat in binding with RAD51, leading to inhibition of RAD51 multimerization. To test this possibility, we compared the gel filtration profile of RAD51 multimerization in the presence of IBR2, B6 or vehicle alone. In the presence of IBR2, the RAD51 elution profile exhibited a major peak consistent with the molecular weight of a monomer, while in the presence of the inactive compound B6 or vehicle alone, the majority of RAD51 formed multimers (Fig 1H), indicating that IBR2, but not B6, can inhibit RAD51 multimerization.

### IBR2 directly and specifically binds RAD51

**Figure 2:**
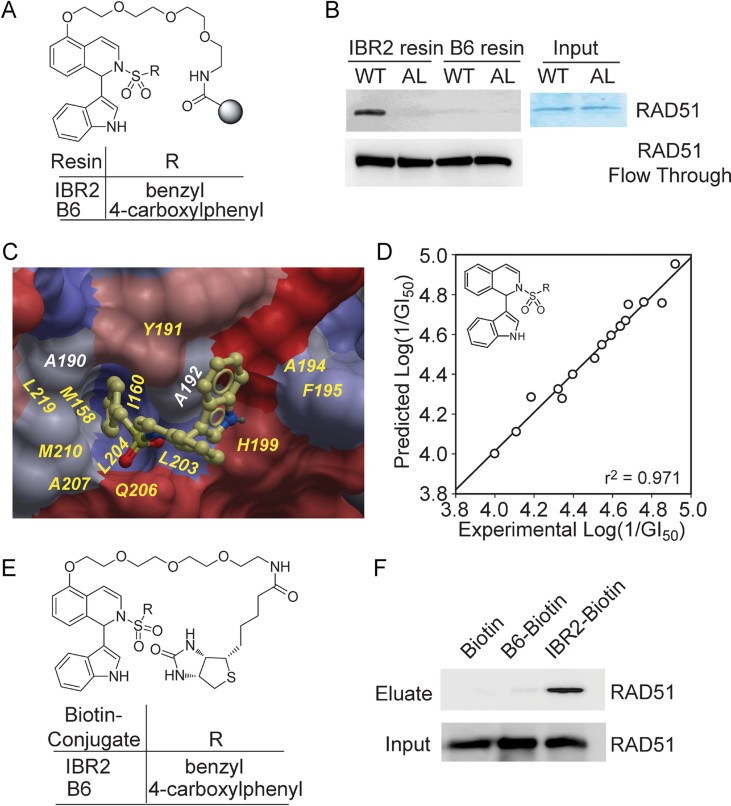
IBR2 directly binds RAD51 in cells Chemical structures of IBR2- and B6-conjugated affi-gel resins.IBR2 affi-gel resin binds to wild-type RAD51 (WT) but not the RAD51 mutated in the binding site (A190/192L, AL). B6 affi-gel resin is negative control.IBR2-RAD51 docking model. IBR2 is shown in ball-and-stick model and coloured by element. RAD51 surface is coloured by hydrophobicity. Residues within 5 Å distance of the docked IBR2 are labelled in yellow; two residues (A190, A192) that are mutated in the AL mutant are labeled in white. Residue numberings are consistent with those in the crystal structure of 1N0W.The quantitative structure–activity relationship of the phenyl moiety on a series of IBR2 analogues (R = benzyl, *m*,*m*-dimethylbenzyl, 4-fluorobenzyl, 4-*tert*-butylbenzyl, phenyl, 4-fluorophenyl, 4-methylphenyl, 4-*tert*-butylphenyl, 4-carboxylphenyl, 4-nitrophenyl, 2-naphthyl, methyl, ethyl, isopropyl, 1-butyl, and phenethyl). To derive the predictive QSAR model, GI_50_ values were correlated with molecular fingerprints using a partial linear regression method and cross validated using a leaving-one-out method. The model was built by the QSAR module in ICM.Chemical structures of the biotin-conjugated IBR2 and B6.RAD51 protein is pulled-down from HeLa cell lysate treated with IBR2-biotin but not B6-biotin using neutravidin resin.

When two alanines (A190, A192) located at the entrance of the multimerization site were mutated to leucines, RAD51 multimerization was blocked, resulting in a constitutively inactive RAD51 mutant (A190/192L, AL; Yu et al, 2003). We reasoned this AL mutant could not bind IBR2. Therefore, we conjugated IBR2 or B6 to affi-gel resins (Fig 2A) and compared their binding affinity with purified wild-type (WT) and AL mutant RAD51. While B6-conjugated affi-gel did not bind to either protein, IBR2-conjugated affi-gel preferentially bound to WT but not the AL mutant RAD51 (Fig 2B), suggesting IBR2 specifically binds to RAD51 through the multimerization pocket on the RAD51 core domain.

Molecular docking suggests that the binding site of IBR2 on RAD51 consists of the following residues: M158, I160, A190, Y191, A192, A194, F195, H199, L203, L204, Q206, A207, M210, L219 (Fig 2C). Blocking of this site using excess BRC peptide inhibits RAD51 multimerization and filament formation, leading to deficiencies in DNA repair (Chen et al, 1998, 1999; Davies et al, 2001). This is the same ‘hot-spot’ site for the interaction with the phenylalanines of BRC4 hairpin (F1524) (Pellegrini et al, 2002) and the RAD51 multimerization motif (F86, equivalent to F144 in *Saccharomyces cerevisiae* Rad51 (Conway et al, 2004) and F97 in *Pyrococcus furiosus* Rad51 (Shin et al, 2003)). Since IBR2 may mimic these phenylalanine residues when binding to RAD51 through its multimerization pocket, the biological activity of IBR2 will be sensitive to structural variations on its phenylsulphonyl moiety. To explore the structure–activity relationship on the phenylsulphonyl moiety, we synthesized a series of compounds sharing the same indolyl-isoquinoline scaffold as in IBR2. We then examined the growth inhibitory effect of these compounds in MCF7 cells. A predicative QSAR model was built and cross-validated based on the correlation between these GI_50_s and the molecular structures using a partial linear regression method (Fig 2D, *r*^2^ = 0.971). We observed loss of growth inhibitory effect with large substituents on the phenyl moiety, suggesting the phenyl moiety of IBR2 is situated in a spatially confined environment on RAD51 protein.

To further demonstrate that IBR2 can directly target RAD51 in a cellular context, we synthesized biotin-conjugated IBR2 and B6 (Fig 2E) for an affinity pull-down assay using HeLa cell lysate. The result indicated that RAD51 was preferentially bound to IBR2-biotin, but not B6-biotin or biotin alone (Fig 2F and Supporting Information Fig S2), suggesting a specific and direct binding between IBR2 and RAD51 in a complex cellular environment.

### IBR2 inhibits RAD51-mediated HR repair

**Figure 3:**
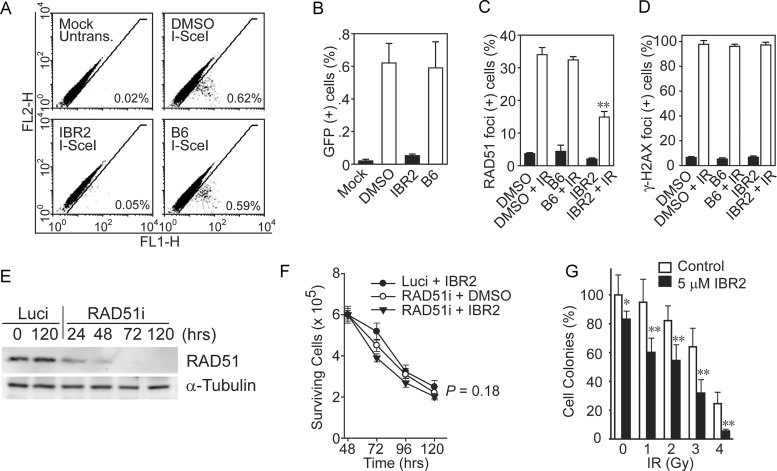
IBR2 inhibits RAD51-mediated homologous recombination repair **A,B.** HR frequency is measured by two-colour fluorescence flow cytometric analysis using HeLa-DR-GFP cells. Fifty thousand events are analysed for each experiment. Representative data are shown in (**A**) for cells without I-SceI transfection (Mock), cells treated with vehicle (DMSO) or 20 µM IBR2 or B6 for 32 h after transient transfection with I-SceI expression vector pCABSce for 4 h, respectively. Percentage of GFP-positive cells is indicated in each panel. FL1-H: forward scatter; FL2-H: side scatter. HR inhibition results from three independent experiments are summarized as means ± SD, as shown in (**B**).**C,D.** Upon IR treatment, IBR2 inhibits the foci formation of RAD51, but not that of γ-H2AX. MCF7 cells are incubated with IBR2 (20 µM) or B6 (40 µM) for 8 h, and then exposed to 8-Gy γ-radiation. After 4 h the irradiated cells are fixed and immunostained with anti-RAD51 or anti-γH2AX antibodies. The percentage of RAD51 (**C**) or γ-H2AX (**D**) foci positive cells is quantified. Note that IBR2 significantly reduced the formation of IR-induced RAD51 foci (*p* = 0.006, *t*-test), but B6 did not (*p* = 0.25, *t*-test).**E,F.** IBR2 is not additive to RAD51 RNAi treatment. HeLa cells were treated with RAD51- or luciferase-RNAi (Luci) for indicated time, and RAD51 level was determined by Western blotting (**E**). IBR2 (15 µM) or DMSO was added 48 h post RNAi treatment. Surviving cells were measured by trypan blue exclusion assay at various time points to derive the survival curves (*p* = 0.18, Two-way ANOVA) (**F**).**G.** IBR2 sensitizes MCF7 cells to γ-irradiation treatment. Clonogenic growth of MCF7 cells after treatment with IBR2 or γ-irradiation or both, expressed as the percentage of control. Statistical significance was determined by student *t*-test (*p < 0.05; **p < 0.01). Each bar represents means ± SD from three independent experiments.

RAD51 multimerization is functionally critical for HR in cells. If IBR2 inhibits RAD51 multimerization, HR rate will be reduced. To test this possibility, we adopted an I-SceI inducible gene conversion assay that measures the DSB repair rate by detecting successful restoration of a fluorescent GFP from the repair substrate DR-GFP (Pierce et al, 1999). DR-GFP, consisting of two nonfluorescent GFP derivatives, SceGFP and iGFP, was first stably integrated into HeLa cells. Upon I-SceI expression to induce DSBs, the GFP positive population resulting from successful recombination was measured by flow cytometry. We then added IBR2 (or B6) to the cells to determine the effects on HR. As shown in Fig 3A and B, the HR rate was significantly reduced after IBR2, but not B6 treatment. Similarly, IBR2 treatment may diminish IR-induced RAD51 foci formation, an event indicative of RAD51-mediated HR in DNA DSB repair. We then treated MCF7 cells with IBR2 or B6 and assessed the nuclear foci formation upon exposure to IR. As shown in Fig 3C and Supporting Information Fig S3, IBR2, but not B6, significantly reduced the formation of IR-induced RAD51 foci (*p* = 0.006), indicating impaired RAD51 assembly during DSB repair. In contrast, both IBR2 and B6 had little effect on the IR-induced foci formation of γ-H2AX, which is phosphorylated at serine 139 by ATM for the recruitment of NBS1, 53BP1and BRCA1 (Celeste et al, 2002; Fig 3D). Taken together, these results suggest that IBR2 specifically inhibits RAD51-mediated HR and diminishes IR-induced RAD51 foci.

If RAD51 is responsible for IBR2's effect in cells, IBR2 treatment will be phenotypically silent in cells depleted of RAD51, which induces significant lethality in cancer cells. To test this possibility, cells were exposed to either RAD51 siRNA (RAD51i) or luciferase siRNA (Luci) alone for 48 h, followed by combination treatment with IBR2 or vehicle, and harvested to determine viability (Fig 3E). As shown in Fig 3F, RAD51 siRNA and IBR2 alone each reduced cell number with similar kinetics, and co-treatment with both agents did not show additive killing effect (*p* = 0.18). These results suggest that IBR2 is phenotypically silent in cells with a RAD51 deficient background.

To further validate that RAD51 is the target of IBR2, we reasoned that when cells are subjected to genotoxic conditions, such as ionizing radiation (IR), their survivals become more dependent on functional RAD51. Hence, we envisioned that IBR2 treatment could lead to increased sensitivity of cells to IR. To test this possibility, we used a low dose of IBR2 (5 µM), which is insufficient to elicit significant RAD51 degradation, and examined its effect on MCF7 cell growth under IR treatment using a colony formation assay. As shown in Fig 3G, treatment with low dose of IBR2 increased the sensitivity of MCF7 cells to IR treatment (0–5 Gy) in a dose-dependent manner, suggesting that the DNA repair function of MCF7 cells upon radiation is compromised by the presence of low dose IBR2.

### IBR2 reduces RAD51 protein levels via proteasome-mediated degradation

**Figure 4:**
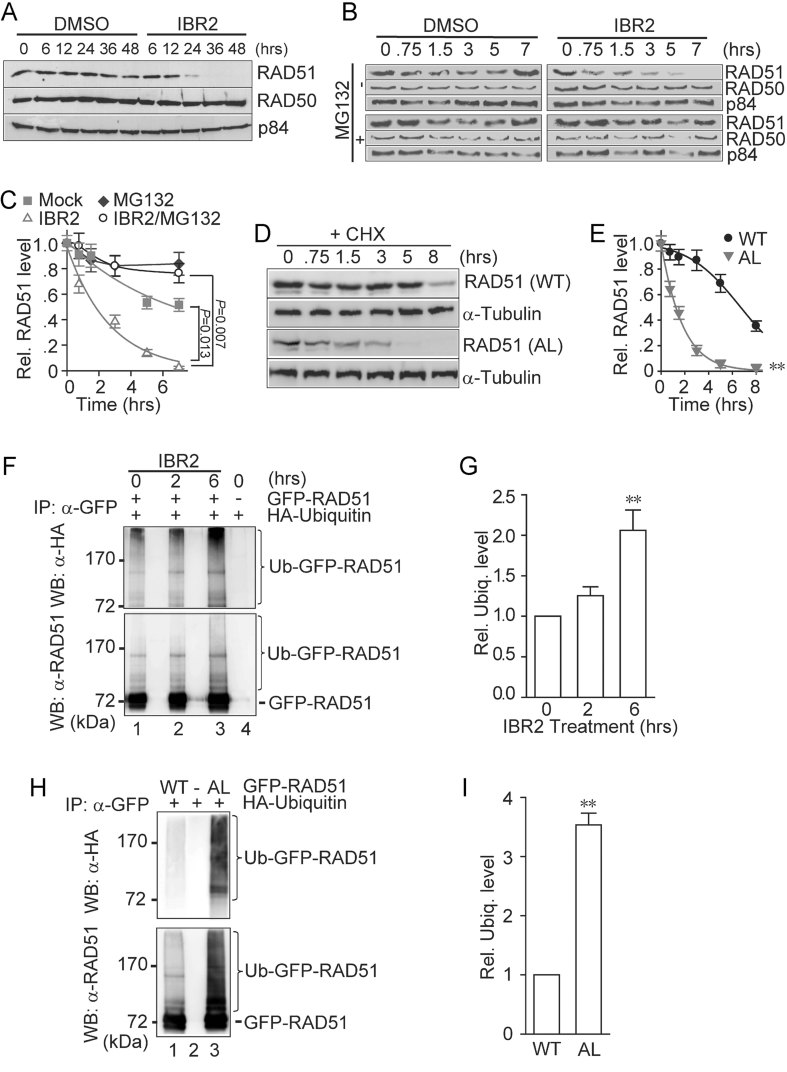
IBR2 reduces RAD51 protein level via proteasome-mediated degradation **A.** IBR2 treatment leads to decreased RAD51 protein level. Cells are incubated with DMSO or IBR2 (20 µM) for indicated time before Western blot analysis. RAD50 and p84 are used as protein level internal control.**B,C.** IBR2 accelerates proteasome-mediated RAD51 degradation. Cells are treated with 50 µg/ml cycloheximide (CHX) alone or together with 20 µM IBR2 and/or 10 µM MG132 before Western blot analysis (**B**). The RAD51 bands are quantified and the relative intensities are plotted in (**C**). The band intensity at 0 h is defined as 1. Each data point represents means ± SD from two independent experiments (*p* = 0.007 and 0.013, Paired *t*-test).**D,E.** GFP-RAD51-wildtype (WT) and GFP-RAD51-A190/192L mutant (AL) half-life assay. Cells expressing RAD51 were treated with CHX, and then harvested at 0–8 h for SDS–PAGE and Western blotting (**D**). RAD51 levels were quantified and plotted against time (Levels at 0 h were normalized to 1) (**E**). Each data point represents means ± SD from two independent experiments (*p* = 0.012, paired *t*-test).**F,G.** Increased RAD51 ubiquitination upon IBR2 treatment. HeLa cells co-expressing GFP-RAD51 (WT) and HA-ubiquitin were treated with IBR2 for indicated times, incubated with MG132 for 1 h, and then harvested for immunoprecipitation. (**F**) Relative RAD51 ubiquitination levels were quantified (**G**). Each bar represents mean ± SD from two independent experiments (*p* = 0.017, *t*-test).**H,I.** Increased ubiquitination in the RAD51 AL mutant. HeLa cells co-expressing GFP-RAD51 (WT or AL) and HA-ubiquitin were treated with MG132 1 h before harvesting cells for immunoprecipitation. Ubiquitinated GFP-RAD51 was analysed by Western blotting (**H**). Relative level of RAD51 ubiquitination was quantified (**I**). Each bar represents mean ± SD from two independent experiments (*p* = 0.011, *t*-test).

To explore the mechanism of how IBR2 inactivates RAD51 function in cells, we first examined RAD51 mRNA level by Q-PCR, upon IBR2 treatment for up to 24 h. As shown in Supporting Information Fig S4, IBR2 treatment did not affect RAD51 on the transcriptional level. We then examined the cellular RAD51 protein level upon IBR2 treatment. While RAD50, ERK and phosphor-ERK levels remained constant throughout IBR2 treatment, RAD51 level decreased in a time-dependent manner (Fig 4A and Supporting Information Fig S5). Moreover, RAD51 stability was measured; and the half-life of RAD51 in IBR2-treated cells was only 1.5 h, compared with 5.5 h in the untreated controls (Fig 4B and C, *p* = 0.013). Since RAD51 degradation is through the proteasome pathway (Kovalenko et al, 1996), IBR2-bound RAD51 may become increasingly susceptible to proteasome-mediated degradation. Indeed, addition of the proteasome inhibitor MG132 to IBR2-treated cells prolonged the half-life of RAD51 (Fig 4C, *p* = 0.007) to a level similar to that of the control protein, RAD50 (Fig 4B), confirming that RAD51 degradation was mediated through the proteasome pathway. Similar results were obtained using another proteasome inhibitor, Lactacystin (Supporting Information Fig S6). Consistently, the half-life of RAD51(AL) mutant was ∼1.7 h; significantly lower than that of the wild-type RAD51 (WT; Fig 4D and E), indicating that the RAD51(AL) mutant, which is unable to form multimers and fails to bind IBR2, was also prone to degradation via the proteasome pathway (*p* = 0.012).

To further confirm that RAD51 degradation induced by IBR2 was via the proteasome pathway, we examined the ubiquitination status of RAD51 upon IBR2 treatment. As shown in Fig 4F and G, IBR2 treatment induced poly-ubiquitination of RAD51 in a time-dependent manner (*p* = 0.017). Consistently, the AL mutant RAD51 constantly showed an elevated level of poly-ubiquitination (Fig 4H and I, *p* = 0.011). These results together indicate IBR2 treatment accelerates RAD51 degradation through the ubiquitin-proteasome pathway.

The initial inhibition of RAD51 multimerization by IBR2 has already impaired RAD51 functions. The subsequent proteasome-mediated protein degradation leads to a continuous deficiency of RAD51, which contributes to growth retardation and cell death of cancer cells (Supporting Information Table S1). Indeed, IBR2 treatment inhibits cell growth in a panel of cancer cell lines, including K562 (GI_50_: 12.5 µM), HeLa (GI_50_: 14.5 µM), MDA-MB-231 (GI_50_: 14.2 µM), MDA-MB-435 (GI_50_: 11.5 µM), MDA-MB-468 (GI_50_: 13.2 µM), MCF7 (GI_50_: 12.3 µM), T47D (GI_50_: 12.1 µM) and HBL100 (GI_50_: 16.0 µM), suggesting a potential use as a broad spectra anti-cancer regimen. Hence we tested the effect of IBR2 on a breast cancer xenograft model, and found IBR2 significantly inhibited the growth of the xenografted tumour in nude mice (Supporting Information Fig S7). Autopsy revealed little or no apparent physiological abnormality in the treated animals.

### IBR2 inhibits cell growth and induces apoptosis in imatinib-resistant T315I-Ba/F3 cells

**Figure 5:**
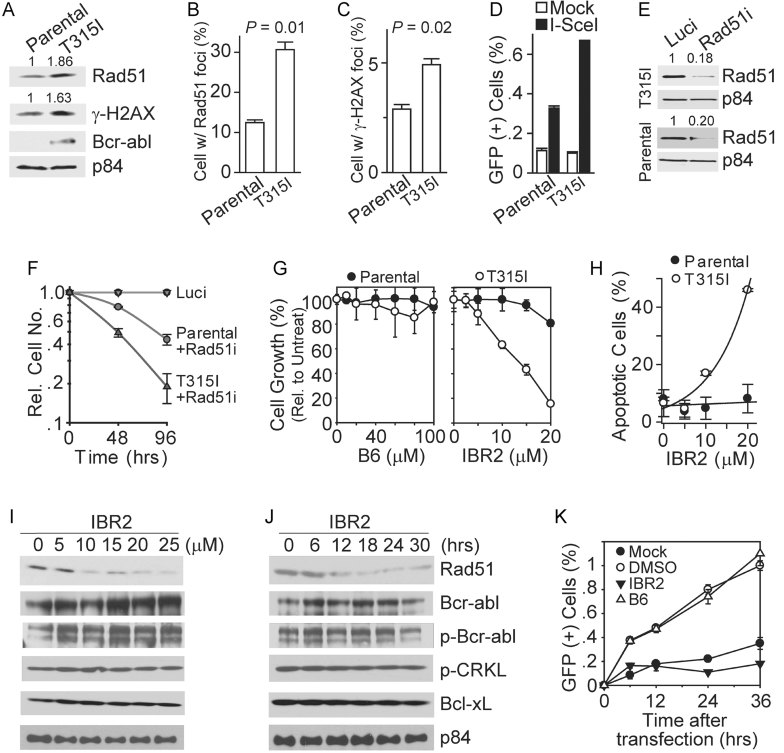
IBR2 inhibits cell growth and induces apoptosis in imatinib-resistant T315I-Ba/F3 cells **A–D.** T315I-Ba/F3 cells (T315I) have a higher basal level of DNA damage response and recombination than parental Ba/F3 cells (Parental), including: increased RAD51 and γ-H2AX protein levels (**A**), increased populations with RAD51 (**B**) or γ-H2AX (**C**) nuclear foci and increased HR rate (**D**). Protein levels were shown by Western blotting (A); nuclear foci positive cells were measured in 500 immunofluorescent stained cells (**B,C**); the basal HR levels in parental cells and T315I cells were based on a DR-GFP/I-SceI recombination assay and measured by two-colour fluorescence analysis of GFP-positive cells. HR frequency (%) was calculated as the percentage of GFP-positive cells. Fifty thousand events were analysed for each experiment, and experiments were performed in triplicate. FL1-H: forward scatter; FL2-H: side scatter (**D**).**E,F.** Knock-down of Rad51 in T315I cells leads to cell growth inhibition. Exponentially growing cells are exposed to Rad51 siRNA or Luci for 48 and 96 h; then Rad51 protein level is analysed by Western blot (**E**). Viable cell numbers are counted and normalized to the Luci treated group to obtain the relative cell numbers; experiments were performed in triplicate (**F**).**G.** IBR2 but not B6 inhibits the growth of T315I cells. Dose–response curves are obtained by XTT assay after 48-h exposure of parental or T315I cells to the indicated concentrations of B6 (left) or IBR2 (right panel). Relative cell growth percentage was obtained by normalizing the cell numbers of treated groups against those of untreated groups (Untreat). Each data point represents means ± SD (*n* = 4).**H.** IBR2 dose-dependently induces apoptotic cell death in T315I cells. Parental and T315I cells are treated with IBR2 at indicated concentrations for 48 h, stained with Annexin V-FITC and PI, and subjected to flow cytometric analysis. Each data point represents means ± SD from three independent experiments.**I,J.** IBR2 reduces Rad51 level in T315I cells in a dose-dependent and time-dependent manner. T315I cells are treated with IBR2 at indicated concentrations for 36 hr (**I**) or 20 µM IBR2 for indicated hours (**J**) before analysis. Whole cell lysates are subjected to Western blot, and probed with Rad51, Bcr-Abl, p-Bcr-Abl, p-CRKL and Bcl-xL antibodies. p84 serves as loading control.**K.** IBR2 inhibits HR in T315I cells. T315I cells are first mock transfected (mock) or transient-transfected with I-SceI expression vector pCABSce for 4 h, followed by treatment with vehicle (DMSO) or 20 µM IBR2 or 40 µM B6 for indicated times (6, 12, 24 and 36 h, respectively). HR frequency is calculated as the percentage of GFP-positive cells and plotted against lapsed time post transfection. Fifty thousand events are analysed for each experiment; and experiments are performed in triplicates.

While IBR2 inhibits the growth of many common cancer subtypes, we sought to examine whether IBR2 is effective in hard-to-treat cancers, such as CML resistant to imatinib (an inhibitor of the tyrosine kinase Bcr-abl encoded by the Philadelphia reciprocal translocation and used to treat CML and GI stromal tumors). We used murine Ba/F3 hematopoietic cells (parental) and Ba/F3 cells expressing the Bcr-abl T315I mutant as a model system. The T315I point mutation renders cells resistant to imatinib, dasatinib and nilotinib, and is observed in about 15% of the imatinib-resistant CML cases (Gorre et al, 2001). The T315I cells exhibit increased proliferation rate (Supporting Information Fig S8), elevated basal expressions of Rad51 and γ-H2AX (Fig 5A) and display increased endogenous DNA damage response, indicated by increased Rad51 and γ-H2AX foci (Fig 5B and C), when compared with parental cells. Furthermore, T315I cells have a nearly twofold increase in basal HR events over parental cells (Fig 5D), suggesting that RAD51 plays an important role in CML, in agreement with previous studies (Skorski, 2002). Consistently, both parental and T315I cell lines exhibited growth inhibition upon depletion of Rad51 by RNAi (Fig 5E), when compared with cells treated with Luciferase RNAi. Importantly, T315I cells were more sensitive to the loss of Rad51 than parental cells (Fig 5F). Similarly, treatment with IBR2 inhibited the proliferation of T315I cells (GI_50_, 12 µM) much more effectively than that of parental cells, while B6 (up to 100 µM) did not inhibit the growth of either cell line (Fig 5G). Since continuous loss of Rad51 protein leads to cell death, the observed cell growth inhibition in T315I cells may result from apoptosis. Therefore, we determined the percentage of apoptotic cells after IBR2 treatment. As shown in Fig 5H, IBR2 induced apoptosis of T315I cells, but not parental cells, in a dose dependent manner, which is correlated with the reduction of Rad51 protein expression (Fig 5I).

To verify that IBR2 also inhibits HR in T315I cells, we used the I-SceI inducible gene conversion assay to measure the HR rates as we did in HeLa cells. The HR rate of T315I cells was significantly reduced upon IBR2 treatment when compared with parental cells (Supporting Information Fig S9). We have also observed a dose-dependent reduction of HR efficiency with increasing IBR2 concentration (Supporting Information Fig S9). This reduction was time-dependent and correlated with the decrease of Rad51 level (Fig 5J and K). Moreover, inhibition of HR by IBR2 occurred prior to complete Rad51 degradation. Inhibition of HR began 6 h after IBR2 treatment and remained so throughout the course of the experiment, suggesting that IBR2-bound Rad51 was first inactivated and then degraded. Because Bcr-abl up-regulates Rad51 activity (Slupianek et al, 2001), we also measured whether IBR2 affects this upstream regulator and found it did not (Fig 5J). Furthermore, IBR2 did not diminish anti-apoptotic molecules such as Bcl-xL (Fig 5J), indicating that the apoptotic effect of IBR2 is unlikely due to inhibition of anti-apoptotic regulators.

### Overcoming CML drug resistance by IBR2 treatment

**Figure 6:**
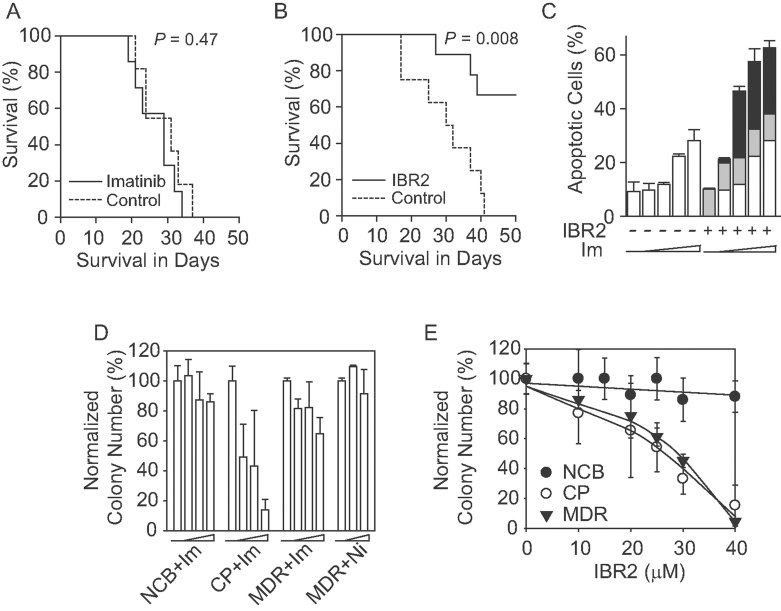
Overcoming drug resistant CML by IBR2 treatment **A,B.** Kaplan-Meier survival analysis of imatinib or IBR2 treated NOD/SCID mice harbouring T315I mutant.**C.** IBR2 synergizes with imatinib in killing human CML cells. K562 cells are treated with imatinib (Im, 0, 0.1, 0.2, 0.4 and 0.6 µM) and IBR2 (15 µM) for 48 h, then stained with annexin V-FITC and PI. Percentages of apoptotic cells are measured by flow cytometry. Results are average of two independent experiments. White bars indicate apoptosis elicited by imatinib alone; grey bars indicate apoptosis elicited by IBR2 alone; and black bars indicate the fraction of apoptosis caused by synergism.**D,E.** Three thousand CD34^+^ cells from cord blood samples or CML patients are treated with Im, nilotinib (Ni), or IBR2 for 96 h. Cells are then plated in semisolid methylcellulose progenitor culture medium for 10–14 days. Experiments are performed in triplicates, granulocyte-macrophage colony forming units (CFC-GM) are quantitated. Normalized colony numbers (%) are presented as means ± SEM for normal cord blood samples (NCB, healthy donor number: *n* = 4), chronic phase-CML samples (CP, patient number: *n* = 5) and multi-drug-resistant samples (MDR, patient number: *n* = 3).**D.** NCB cells are not sensitive to imatinib (Im, 0, 0.5, 1 and 5 µM); CP cells are sensitive to imatinib (Im, 0, 0.5, 1 and 5 µM); and MDR cells are resistant to both imatinib (Im, 0, 0.5, 1 and 5 µM) and nilotinib (Ni, 0, 1 and 5 µM).**E.** Both CP and MDR, but not NCB, cells are sensitive to IBR2 in a dose-dependent manner.

To show IBR2 has therapeutic potential, we intravenously injected Non-Obese Diabetic/Severe-Combined Immunodeficient (NOD/SCID) mice (Shah et al, 2004) with T315I cells followed by treatment with either IBR2 or imatinib alone for 20 days. Compared with the control (vehicle alone), daily treatment with 125 mg/kg imatinib showed no significant improvement in animal survival (Fig 6A, *p* = 0.4711). In contrast, the IBR2 (100 mg/kg) treated groups showed significantly prolonged survival (Fig 6B, *p* = 0.0079).

To extend the mouse model to human leukaemia, we first used K562, which is a BCR-ABL positive cell line derived from a CML patient in blast crisis and are resistant to most forms of chemotherapy. Treatment with IBR2 alone at 15 µM induced ∼10% apoptosis; whereas 0.1, 0.2, 0.4 and 0.6 µM imatinib treatment induced apoptosis at about 9.7, 11.8, 22.3 and 28.1%, respectively. Combined IBR2-imatinib treatment resulted in substantial increases of apoptotic percentages, to 21.2, 46.5, 57.5 and 62.6%, respectively (Fig 6C), suggesting that co-treatment of IBR2 and imatinib in K562 has a synergistic effect on cell killing as indicated by the combination index (CI < 1, Supporting Information Fig S10). Similar synergistic effect was observed in the treatment of Ba/F3 cells bearing wild-type Bcr-Abl (p210) using IBR2 and imatinib (Supporting Information Fig S11). Next, we examined the efficacy of IBR2 on primary hematopoietic progenitor cells using colony-forming cell (CFC) assays. As shown in Fig 6D, when exposed to various concentrations of imatinib (0.5–5 µM), normal cord blood cells (NCB, as control) and advanced stage multi-drug-resistant CML cells (MDR) showed minimal inhibition, while the colony formation ability of chronic phase cells (CP) was inhibited in a dose-dependent manner. MDR cells were also resistant to nilotinib (Ni, inhibition < 9%), which is a second generation BCR-ABL kinase inhibitor. We then treated cells with IBR2 at indicated concentrations for 96 h and determined the effects of drug exposure on CFC numbers. As shown in Fig 6E, similar to Imatinib, IBR2 did not significantly inhibit the growth of NCB cells (<10% up to 40 µM IBR2), whereas 10, 20, 30 and 40 µM IBR2 suppressed the growth of CP cells to approximately 20, 35, 65 and 85%, respectively. Interestingly, 10, 20, 30 and 40 µM IBR2 significantly inhibits the growth of imatinib-resistant MDR cells to approximately 15, 25, 60 and 90%, respectively. No significant inhibitory effect of IBR2 and imatinib were observed on CD34^+^ normal bone marrow cells (Supporting Information Fig S12). These results together suggest that IBR2 may be used as anti-leukaemia agent in the treatment of CML patients who have developed resistance to multiple tyrosine kinase inhibitors.

## DISCUSSION

Up-regulation of RAD51 stimulated by fusion tyrosine kinases, *e.g.* BCR-ABL, confers resistance to chemotherapy-induced apoptosis. As we have shown here, IBR2 effectively induces apoptosis in the imatinib-resistant T315I cells, and extends the lifespan of the T315I xenograft models. IBR2 also inhibits the growth of primary imatinib-resistant CML cells isolated from patient samples. Given the unstable genomic background of cancer cells, tyrosine kinases are prone to secondary mutations, leading to drug-resistance issues (Deininger et al, 2005; Gorre et al, 2001; Nardi et al, 2004). Mutations in BCR-ABL were found in the imatinib/nilotinib resistant patient samples used in this study. In contrast, mutation in RAD51 is extremely rare. Taken together, targeting aberrantly elevated RAD51 function may provide an alternative way for treating CML patients resistant to tyrosine kinase inhibitors.

Cancers, including acute lymphocytic leukaemia, acute/chronic myeloid leukaemia, pancreatic cancer, breast cancer, etc, depend on elevated RAD51 functions and enhanced HR rate for their proliferation, survival and drug resistance capabilities (Raderschall et al, 2002; Skorski, 2002; Slupianek et al, 2001; Vispe et al, 1998). This is consistent with our observations that T315I cells have elevated basal level HR activity over parental cells (Fig 3B and C). Moreover, upon IBR2 treatment, the HR activity in T315I was reduced to a level comparable to that in parental cells, while the latter was relatively insensitive to the treatment, suggesting a potential therapeutic window (Supporting Information Fig S9). Consistently, without genotoxic stress, the survival of non-proliferating somatic cells in mature animals does not require as much HR activity (Luo et al, 2009), and therefore are less sensitive to RAD51 inhibition. Indeed, the body weight of nude mice is not affected by Rad51 inactivation via daily IBR2 treatment (Supporting Information Fig S13), and IBR2 has no significant inhibitory effect on CD34^+^ normal bone marrow cells (Supporting Information Fig S12), suggesting that inhibition of RAD51 can be tumor-selective to a certain extent. Hence, inhibition of RAD51 functions may provide an effective approach to cancer intervention, as also shown by earlier studies using RAD51 RNAi or antisense RNA (Sak et al, 2005).

Developing small molecules to disrupt or modulate DNA repair machinery has recently received increasing attention (Bryant et al, 2005; Connell et al, 2004; Dupré et al, 2008). Peptide based approaches have been explored for developing RAD51 inhibitors (Chen et al, 1999; Connell et al, 2004). As we report here, IBR2 inhibits RAD51 multimerization and renders RAD51 more prone to proteasome-mediated protein degradation. Without IBR2, RAD51 can form multimers when the cells need active HR functions. When treated with IBR2, the multimerization capacity of RAD51 is greatly inhibited, leading to an increased sensitivity of RAD51 to proteasome mediated protein degradation; a similar phenomenon has been observed in the shortened half-life of the constitutively inactivated AL mutant of RAD51. Taken together, IBR2 represents the first small molecule that directly binds RAD51, disrupts RAD51 multimerization, inactivates RAD51 functions both *in vitro* and *in vivo*, which suggests a novel entry point for developing potential therapies in compliment to current treatments for cancer.

Although IBR2 has shown a promising anti-tumor activity in the current cancer model, one should be aware that RAD51 is also an essential factor for normal cells. By exploiting the ‘addiction’ to RAD51 of certain cancers that are under genotoxic stresses, we are optimistic that a therapeutic window can be available for tumor-specific killing. To define this therapeutic window and avoid severe toxicity to normal cells, however, will require continuous effort to develop IBR2 derivatives that are more compatible with preclinical and clinical testing.

## MATERIALS AND METHODS

### General reagents

Compound library was from Nanosyn. IBR2, B6, IBR2-conjugated and B6-conjugated affi-gel resins, and biotin-conjugated IBR2 and B6 were synthesized and characterized in the Synthesis Facility, UC Irvine (Supporting Information). Imatinib mesylate and dasatinib were from Cancer Center Pharmacy, UC Irvine. NTA and CM5 sensor chips were from Biacore. Recombinant His-RAD51 fusion protein, untagged wild-type and A190/192L mutant RAD51 were purified to near homogeneity as previously described (Sung & Robberson, 1995). Recombinant GST-BRC fusion protein (containing BRCA2-1005-1040, fused with GST at its N-terminal) was purified to near homogeneity as previously described (Chen et al, 1998). Human cancer cell lines were maintained in high-glucose Dulbecco Modified Eagle Medium (DMEM; Invitrogen) supplemented with 10% foetal bovine serum (FBS). Ba/F3 cells stably transfected with full-length T315I Bcr-abl mutant (from Dr. C. Sawyers, MSKCC) and K562 cells (American Type Culture Collection) were maintained in RPMI 1640 supplemented with 10% FBS, 50 units/ml penicillin G and 50 µg/ml streptomycin at 37°C in 5% CO_2_. Ba/F3 parental cells were cultured under the same condition, with addition of 10 ng/ml murine IL-3 (R&D systems). SDS–PAGE and immunoblot analyses were performed as described previously (Chen et al, 1999). Antibodies used were: anti-RAD51 (rabbit), -RAD50 (mAb 13B3), -p84 (mAb 5E10) and β-actin (GeneTex). Densitometry quantification was done by Labworks 4.5.

### DNA plasmids, retrovirus and siRNA

RAD51 cDNA was engineered into the pQCXIP retroviral plasmid (RAD51 in frame fused with an N-terminal EGFP from pEGFP-C2 vector) and pET28 bacterial expression plasmids. Mutant RAD51 was made using the Quick change mutagenesis kit (Stratagen). Retrovirus was packaged in the 293GP2 cells with cotransfection of pQCXIP-GFP-RAD51 and pVSVG plamids using a standard procedure (Clontech). RAD51 siRNA was transfected into cells by Lipofectamine 2000 (Invitrogen) for adherent cells or the nucleofection kit (Lonza) for suspension culture (parental and T315I Ba/F3 cells). RNAi sequences are GAGCUUGACAAACUACUUC for human RAD51, and GCGAUGUCCUAGAUAAUGU for mouse Rad51.

### Reverse yeast two-hybrid screening

TetR/NCB, containing BRC repeats 1-4 (BRCA2-927-1596; Chen et al, 1998), was constitutively expressed; AD/RAD51 was expressed under GAL1 promoter. Yeast was grown in a galactose medium containing 5-FOA. The assay was performed on 96-well plates with 10 µM compounds in 100 µl total volume. Compounds that disrupt the BRC-RAD51 interaction were identified by measuring yeast viability.

### Molecular modelling

RAD51 coordinates were from PDB (Accession No: 1N0W). RAD51 residues containing atoms of 5 Å distance from the BRC4 peptide in 1N0W was designated as potential binding site for docking, including M158, Y159, I160, F167, P168, L171, S183, V185, L186, D187, N188, V189, A190, Y191, A192, R193, A194, F195, H199, Q202, L203, L204, Y205, Q206, A207, S208, A209, M210, V212, E213, Y216, L219, R247, R250, M251, L252, R254, L255, E258, F259. Structures of small molecules were generated and optimized and molecular docking was performed using ICM Pro (Molsoft), following standard procedures as described by the software manual, using default docking parameters at thoroughness = 5. Docked conformations with RMSD < 2 Å were considered acceptable, and the lowest energy conformation was shown.

### Surface plasmon resonance binding assay

Surface plasmon resonance assays were performed at 22.5°C in 1×HBSD buffer (10 mM Hepes, 150 mM NaCl, 0.1% DMSO, pH7.5) on Biacore3000 (GE). The glutathione sensor chips were prepared from CM5 chips following standard amine-coupling protocol. The NTA or glutathione sensor chips were used to capture His-RAD51 or GST-BRC, respectively. The capture level was about 130 resonance units at a flow rate of 5 µl/min. For sequential binding assays, chips were pre-treated with IBR1/2 or B6 (1 µM) or mock-treated (buffer only, used for positive binding control) by a single injection (5 µl/min for 1 min), and then exposed to proteins (50 µg/ml, 5 µl/min for 1 min). BSA was used as negative binding control. The retained resonance units (RU) were recorded and averaged from triplicates. For competition assays (Vassilev et al, 2004), various concentrations of compounds IBR2 or B6 were incubated with 50 µg/ml GST-BRC at 25°C for 15 min prior to use. Relative binding percentages were calculated with respect to the binding in the absence of compound and averaged from two independent experiments.

### Gel filtration

A mixture of RAD51 (3.2 µg) and small compound (molar ratio 1:10) was incubated for 15 min at 37°C, supplemented with buffer (50 mM triethanolamine-HCl [pH7.5], 0.5 mM Mg(OAc)_2_, 1 mM DTT, 2 mM ATP and 100 µg/ml BSA, total volume 20 µl) and incubated for 15 min. The mixture was loaded onto a 2.4 ml Superdex 200 PC 3.2/30 column (Pharmacia) equilibrated with the same buffer as previously described (Davies et al, 2001). Fractions (50 µl) were collected and 0.5 µl of each fraction was blotted onto PVDF membrane. RAD51 was detected using anti-RAD51 antibody (mAb 14B4, GeneTex).

### HR assay

As previously described (Pierce et al, 1999), cells were transfected with 3 µg pDR-GFP (courtesy of Dr. Andrew J. Pierce) with FuGENE6 (Roche) and stable colonies were selected with 1.0 µg/ml puromycin (Sigma). Single-copy stable clones were screened by southern blot, for which the DNA probe was prepared using the 0.8 kb HindIII fragment released from plasmid pDR-GFP. The single copy clone yielded one 0.8 kb and one >5 kb positive fragment (Supporting Information Fig S14). Four hours after transfection (FuGENE6) with the I-SceI expression vector pCBASce, cells were treated with compounds or vehicle (DMSO). Cells were then trypsinized and subjected to flow cytometry. Two-colour fluorescence analysis revealed the percentage of GFP-positive cells scored out of 50,000 viable events.

### IBR2-RAD51 affinity binding and pull-down assays

The affinity binding of wild-type and mutant RAD51 with compound-conjugated affi-gel resins was performed as previously described (Wu et al, 2008). For pull-down assays, HeLa cell extract was clarified with neutravidin-resin (Invitrogen) and incubated with compound-biotin conjugates for 2 h; then neutravidin-resin was added and the mixture was shaken for 1 h. The resin was then collected, and washed with binding buffer (1× PBS, 50 resin volumes) and 30 µM B6-linker conjugate in the binding buffer (30 resin volumes). Finally, the resin was eluted with corresponding compound (30 µM IBR2 or B6). The eluates were subjected to SDS–PAGE and Western blot analysis.

### RAD51 stability assay

Cells treated with IBR2 or transfected with GFP-RAD51 for 24 h were treated with cycloheximide, and harvested at various time points for Western blot analysis.

### Ubiquitination assay

The assay was performed following a similar protocol (Poole et al, 2006). Cells were cotransfected with GFP-RAD51 and HA-ubiquitination expression plasmids, and were treated with IBR2 24 h later. After 0–6 h of IBR2 treatment, MG132 (20 µM) was added for 2 additional hours. Cells were directly lysed in 2% SDS plus 5–10 mM NEM and protease inhibitors, sonicated and clarified by spinning. Cell extract was then diluted with TBSN (40 mM tris pH7.4, 100 mM NaCl, 0.1% NP40) for immunoprecipitation assay.

### Nuclear foci formation assay

For IR-induced foci, MCF7 cells were treated with IBR2 (20 µM) or B6 (40 µM) for 8 h and then exposed to 8-Gy γ-radiation. Cells were incubated for additional 4 h, and then fixed with 4% paraformaldehyde in PBS containing 0.5% Triton X-100. The fixed cells were stained with RAD51 antibody (mAb 1F5) or γ-H2AX antibody (Upstate Biotechnology). RAD51 and γ-H2AX foci positive cells (≥5 foci) were subsequently counted as described (Chen et al, 1999). Thousand nuclei per sample were counted to determine the percentage of foci formation. The γ-H2AX and Rad51 nuclear foci formation was examined in T315I-Ba/F3 cells following immunofluorescent staining with respective polyclonal antibodies, as previously described (Zhong et al, 1999). Five hundred cells were assessed for each group.

### Clonogenic survival assay

Cells were seeded at 1 × 10^3^ to 3 × 10^3^ in 10-cm dishes for 24 h, and then treated in triplicates with DMSO, IBR2 (15 µM) or B6 (40 µM) for 2 weeks. Media was replenished every 4 days. The cells were then fixed and stained with 2% methylene blue in 50% v/v methanol. Colonies consisting of more than 50 cells were counted.

### Cell proliferation assay

Exponentially growing cells were seeded into 96-well plates (5000 cells/well), followed by immediate addition of compounds (serial dilutions). After 48-h treatment, cell viability was determined using the XTT cell proliferation assay kit (Roche). Untreated cells were used as controls and complete medium without cells was used to measure the background signal. The absorbance was measured with a Multiskan Ascent microplate reader (Thermo Electron Corp) at 492 nm.

### Apoptosis assay

Annexin V-FITC and propidium iodide (PI, BD Pharmingen) were used to quantify the percentage of apoptotic cells. After drug treatment, cells were collected and resuspended in 100 µl binding buffer (10 mM Hepes, pH 7.4; 140 mM NaCl; 2.5 mM CaCl_2_) at a concentration of 10^7^ cells/ml, then 5 µl of Annexin V-FITC and 1 µl of PI (50 µg/ml) were added. Cells were gently vortexed and incubated for 15 min at room temperature in the dark. An additional 400 µl of binding buffer was added to each tube and the samples were analysed by flow cytometry.

### Survival analysis of Ba/F3-T315I mouse model

Experiments were performed and mice with disease were sacrificed according to the guidelines of the UCI Animal Research Committee. 10^6^ Ba/F3 cells harbouring T315I Bcr-abl mutant were injected into the tail vein of female NOD/SCID mice (7–8 mice per group, 6–7 weeks of age, Jackson Lab.). After 3 days, mice were treated once daily (i.p.) for 20 days with vehicle A (15% DMSO, 20% Tween 20, 10% PEG400, 55% saline) or 100 mg/kg IBR2 (formulated in vehicle A), or 125 mg/kg imatinib (two divided doses formulated in 0.05% methylcellulose) via gavage. Survival analysis was performed using the Kaplan-Meier method and statistical significance was determined using the log rank test.

### Colony forming cell (CFC) assay

Heparin-treated bone marrow samples were obtained from patients with CML and cord blood samples with informed consent, following guidelines approved by the Institutional Review Board of the City of Hope National Medical Center. CD34^+^ normal bone marrow cells were from Stemcell Technologies. CD34^+^ progenitor cells were selected as described (Holtz et al, 2002). CD34^+^ cells were cultured in Iscoves modified Dulbecco medium (IMDM; Gibco), supplemented with 20% FBS, 200 pg/ml granulocyte-macrophage colony-stimulating factor, 1 ng/ml granulocyte colony-stimulating factor, 200 pg/ml stem cell factor, 50 pg/ml leukaemia inhibitory factor, 200 pg/ml macrophage inflammatory protein α, and 1 ng/ml interleukin 6, and various concentrations of compounds of interest. Cells were cultured for 96 h at 37°C in a humidified atmosphere with 5% CO_2_, after which cells were harvested, washed with IMDM and subjected to CFC assay as described (Holtz et al, 2002).

For more detailed Materials and Methods see the Supporting Information.

## Author contribution

PLC designed and performed the high-throughput screening. LZ, XL, GW, GL, HK, CFC, CMH and EG designed and performed cellular experiments. LZ designed and performed animal studies. JZ and XLQ designed and synthesized the compounds. GW performed the compound pull-down and ubiquitination assays. JZ performed the molecular modelling, gel filtration assay and in vitro binding assays. RB supervised the experiments on CD34^+^ progenitor cells. ARC provided intellectual input to the chemical and modelling studies. WHL and PLC provided critical input to the overall research direction. JZ and WHL wrote the paper with input from all co-authors.

**The paper explained**

PROBLEM:

Elevated RAD51 activity is required for cancer cells to proliferate and survive, in response to the increased level of DNA damage in cancers. Therefore, the often-observed hyperactivity of RAD51 in tumour cells is a clinically relevant problem, and drugs able to inhibit or attenuate RAD51 activity might offer novel treatment options for difficult-to-treat malignancies, either as a mono-treatment or in conjunction with known agents.

RESULTS:

This study demonstrates that a newly identified RAD51 inhibitor is capable of direct binding with RAD51 on its multimerization pocket, leading to the disruption of RAD51 multimerization and subsequently the degradation of RAD51 via the proteasome pathway. The compound inhibits cancer cell survival, which is consistent with the siRNA-mediated RAD51 knock-down effect. The drug effect can be abrogated when RAD51 is knocked down by siRNA, suggesting the mode of action of the compound is specific to RAD51. The potential value of RAD51 inactivators as agents for treating cancers is further supported by studies demonstrating that injection of the RAD51 inhibitor IBR2 greatly increased life spans in a mouse CML model, and that treatment of IBR2 inhibited the proliferation of CD34^+^ progenitor cells isolated from late stage CML resistant to tyrosine kinase inhibitors.

IMPACT:

This work identifies small molecule inhibitors specifically targeting RAD51 as a novel pharmacological tool to inhibit growth and induce apoptosis in cancer cells and suggests a broad-spectrum strategy in treating difficult-to-treat cancers.

## Supplementary material

As a service to our authors and readers, this journal provides supporting information supplied by the authors. Such materials are peer reviewed and may be re-organized for online delivery, but are not copy-edited or typeset. Technical support issues arising from supporting information (other than missing files) should be addressed to the authors.
