# Supplementary material for: A novel small molecule RAD51 inactivator overcomes imatinib-resistance in chronic myeloid leukaemia
Source: EMBO Mol Med. 2013 Jan 22;5(3):353–65. doi: 10.1002/emmm.201201760 (PMC3598077; doi:10.1002/emmm.201201760)
Supplement: Supplementary file 1 [file emmm0005-0353-SD1.pdf]

A novel small molecule RAD51 inactivator overcomes imatinib-resistance in chronic myeloid leukemia

Jiewen Zhu, Longen Zhou, Guikai Wu, Heiko Konig, Xiaoqin Lin, Guideng Li, Xiao-Long Qiu, Chi-Fen Chen, Chun-Mei Hu, Erin Goldblatt, Ravi Bhatia, A. Richard Chamberlin, Phang-Lang Chen & Wen-Hwa Lee

Corresponding author: Wen-Hwa Lee, University of California, Irvine

Review timeline:

	Submission date:	19 July 2012
	Accepted	27 November 2012

Transaction Report:

No Peer Review Process File is available with this article, as the authors have chosen not to make the review process public in this case.

© EMBO
