# Supplementary material for: A novel small molecule RAD51 inactivator overcomes imatinib-resistance in chronic myeloid leukaemia
Source: EMBO Mol Med. 2013 Jan 22;5(3):353–65. doi: 10.1002/emmm.201201760 (PMC3598077; doi:10.1002/emmm.201201760)
Supplement: Supplementary file 2 [file emmm0005-0353-SD2.pdf]

Supplementary Information

A novel small molecule RAD51 inactivator overcomes imatinib-resistance in chronic myeloid leukemia (Zhu *et al*)

Table of Contents:

SFigure 1. ……………………………………………………………………*Page 2*
IBR analogues in the screened library.
SFigure 2. ……………………………………………………………………*Page 3*
IBR2 binds RAD51 in cells.
SFigure 3. ……………………………………………………………………*Page 4*
IBR2 inhibits IR-induced RAD51 foci formation in MCF7 cells.
SFigure 4. ……………………………………………………………………*Page 5*
IBR2 does not affect RAD51 mRNA level.
SFigure 5. ……………………………………………………………………*Page 6*
IBR2 induces RAD51 degradation but does not affect ERK/pERK levels.
SFigure 6. ……………………………………………………………………*Page 7*
IBR2 induced RAD51 degradation is blocked by Lactacystin.
SFigure 7. ……………………………………………………………………*Page 8*
The inhibitory effect of IBR2 on the growth of tumor xenografts in nude mice.
SFigure 8. ……………………………………………………………………*Page 9*
T315I cells showed increased proliferation rates compared to parental cells.
SFigure 9. ……………………………………………………………………*Page 10*
IBR2 dosage-dependently inhibits HR in T315I but not parental cells.
SFigure 10. ……………………………………………………………………*Page 11*
Synergistic effect of IBR2 and Imatinib in killing K562 cells.
SFigure 11. ……………………………………………………………………*Page 12*
IBR2 and Imatinib synergistically induce apoptosis in P210 Ba/F3 cells.
SFigure 12. ……………………………………………………………………*Page 13*
IBR2 and Imatinib do not inhibit the growth of $CD34^+$ normal bone marrow cells.
SFigure 13. ……………………………………………………………………*Page 14*
Body weight of nude mice is not affected by IBR2 treatment.
SFigure 14. ……………………………………………………………………*Page 15*
Screening for cells with a single-copy DR-GFP HR reporter.
STable 1. ……………………………………………………………………*Page 16*
Cell cycle progression of IBR2 treatment in MCF7 cells.
Supplementary Methods: ……………………………………………………*Page 17-27*

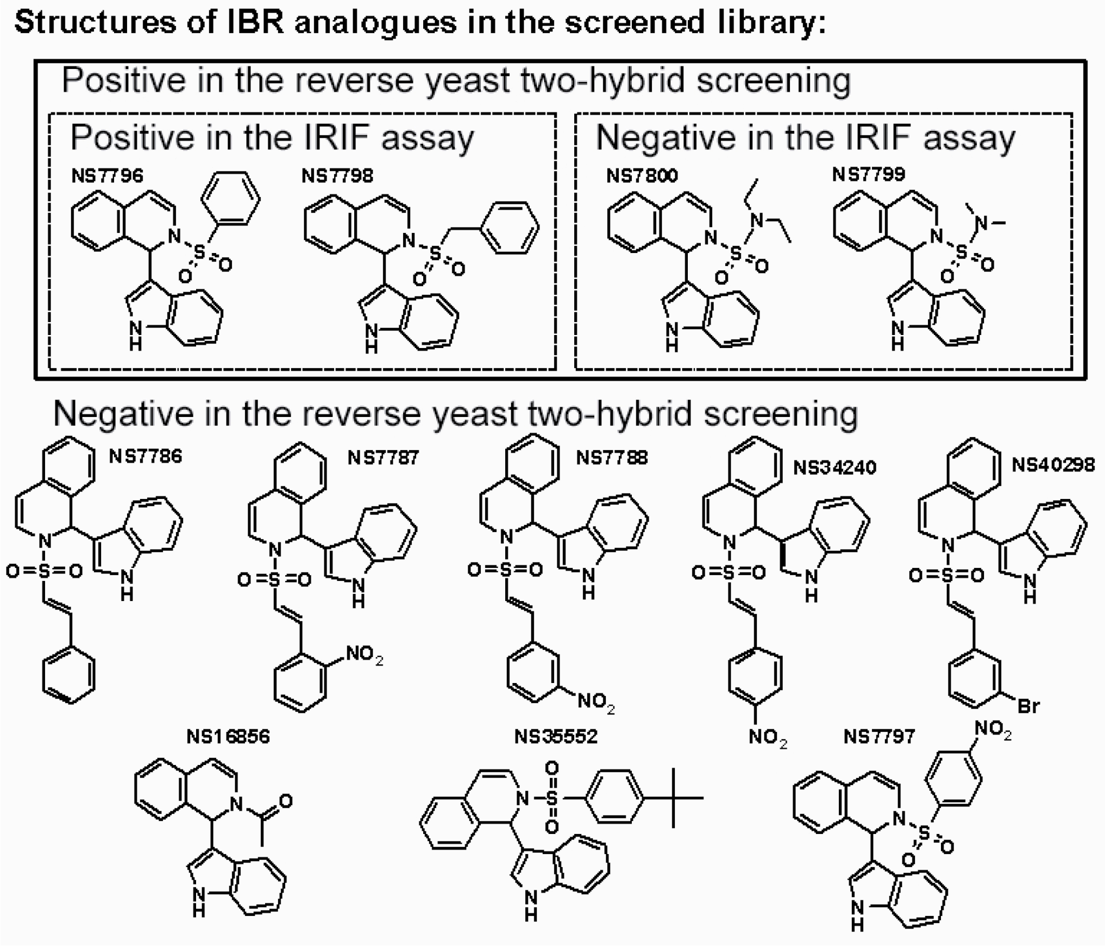

SFigure 1. IBR analogues in the screened library. IBR analogues in the initial reverse yeast two-hybrid screening. Compounds consisting of a core structure containing the isoquinoline and indolyl ring systems are shown. Two compounds, NS7796 (IBR1) and NS7798 (IBR2), were positive in both the yeast two-hybrid screening and IR-induced Rad51 foci formation assay. Two compounds, NS7800 and NS7799, were positive in the yeast two-hybrid screening, but negative in the Rad51 foci formation assay. The remaining compounds were negative in the yeast two-hybrid screening.

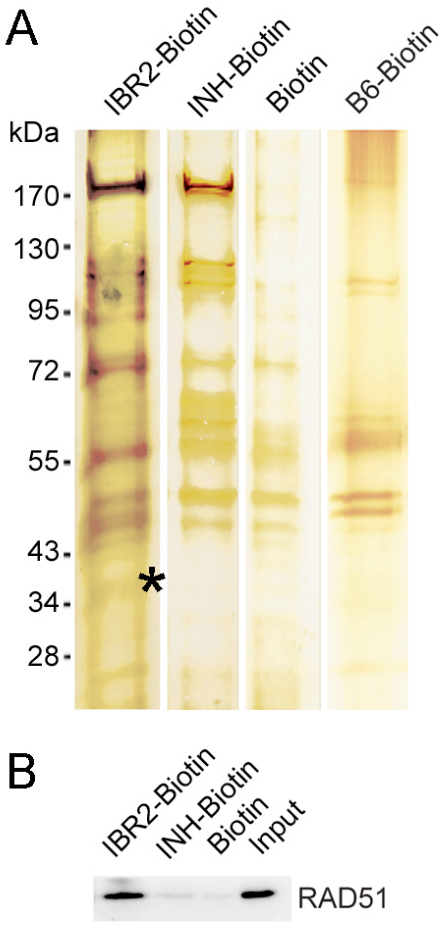

SFigure 2. IBR2 binds RAD51 in cells. (A) Silverstaining of affinity pull-down assays. Note that RAD51 was detected in the IBR2-biotin precipitates, but not in any of the negative control reactions Star (*) indicates RAD51 protein band. **(B)** RAD51 protein is pulled down from HeLa cell lysate treated with biotin-conjugated IBR2, but not with biotin alone or biotin-conjugated INH, (a structurally unrelated compound as we previously described (Wu et al, 2008)), using neutravidin resin. Similar result was obtained using T315I cells.

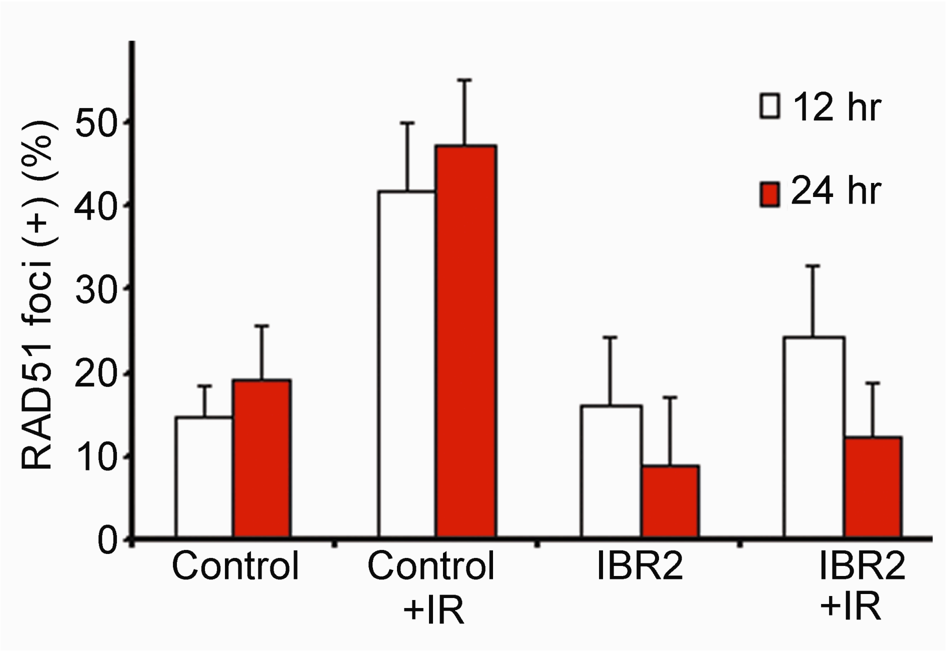

SFigure 3. IBR2 inhibits IR-induced RAD51 foci formation in MCF7 cells. MCF7 cells are incubated with or without IBR2 (20 μM) for 8 hours, and then exposed to γ-radiation (20Gy). After 4 hours the irradiated cells are fixed and immunostained with anti-RAD51 antibodies. The percentage of RAD51 foci positive cells is quantified and shown as means ± s.d.

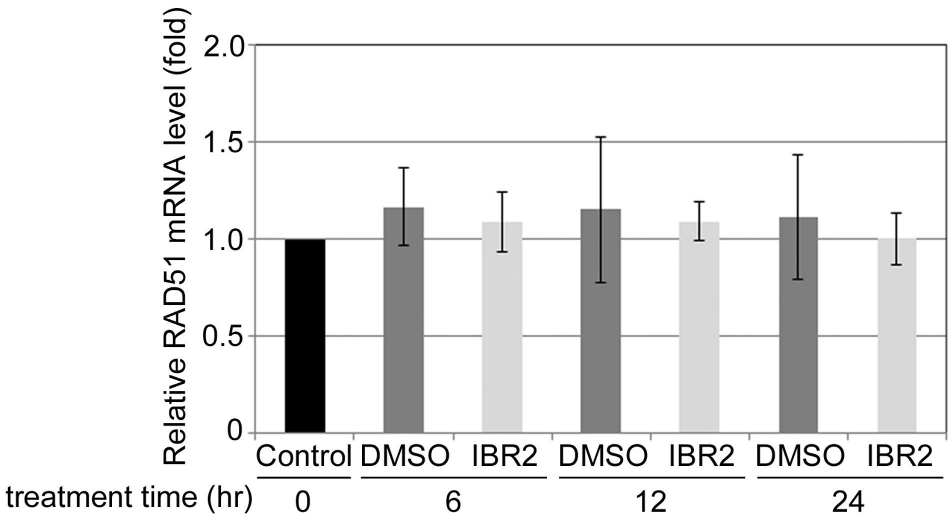

SFigure 4. IBR2 does not affect RAD51 mRNA level. HeLa cells were treated with IBR2 (20 μM) for 6, 12, and 24 hrs; and the cell lysates were prepared for Q-PCR to determine RAD51 mRNA level. Bars represent means ± s.d. from three independent experiments.

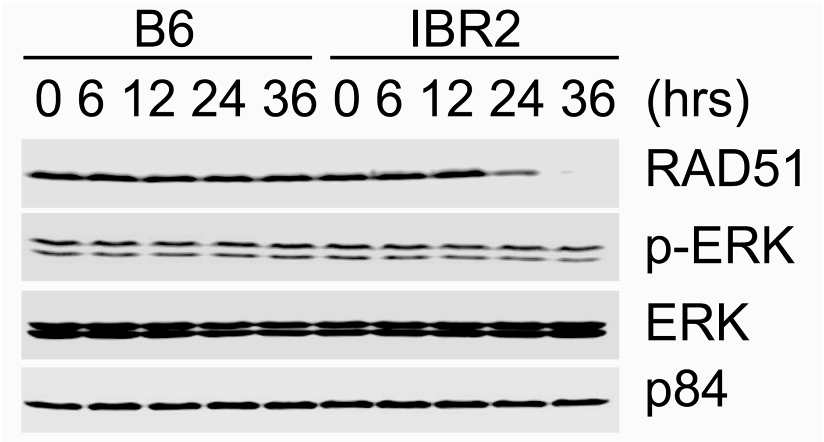

SFigure 5. IBR2 induces RAD51 degradation but does not affect ERK/pERK levels. MCF7 cells were incubated with B6 (100 µM) or IBR2 (20 µM) for the indicated hours and then subjected to Western blot analysis. p84 was used as a protein level internal control.

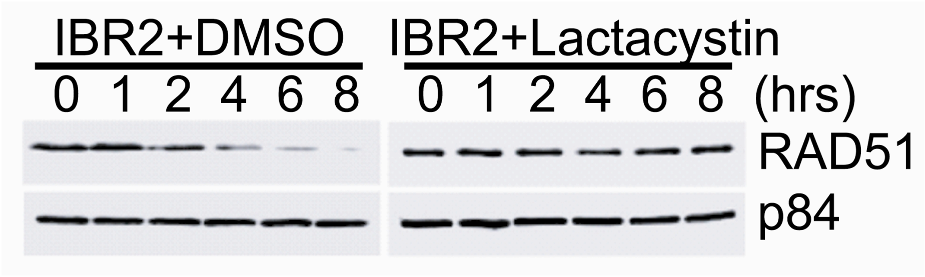

SFigure 6. IBR2 induced RAD51 degradation is blocked by Lactacystin. HeLa cells were treated with 50 μg/ml cyclohexamide with IBR2 (20 μM) and/or Lactacystin (10 μM) for indicated hours and then subjected to SDS-PAGE and Western blotting. p84 was used as a protein level internal control.

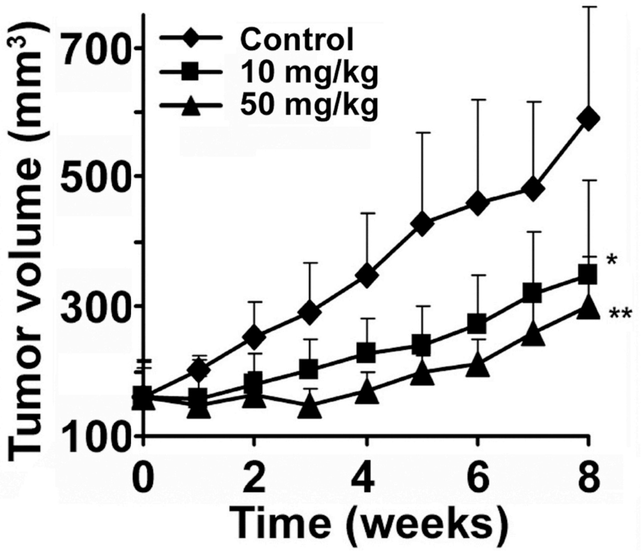

SFigure 7. The inhibitory effect of IBR2 on the growth of tumor xenografts in nude mice. 5×10^6 MDA-MB-468 cells in 100 µl of PBS were injected into mammary fat pad of 6-8 week old athymic female BALB/c-nude mice (nu/nu) (Charles River Laboratories). After tumors grew to 160 mm^3 (day 24), mice were randomized into three groups (n=8 per group) to receive intraperitoneal injection of IBR2 (10 or 50 mg/kg) or vehicle (15% DMSO, 20% Tween 20, 10% PEG400, 55% saline) every day for 8 weeks. *: $P < 0.05$, **: $P < 0.01$.

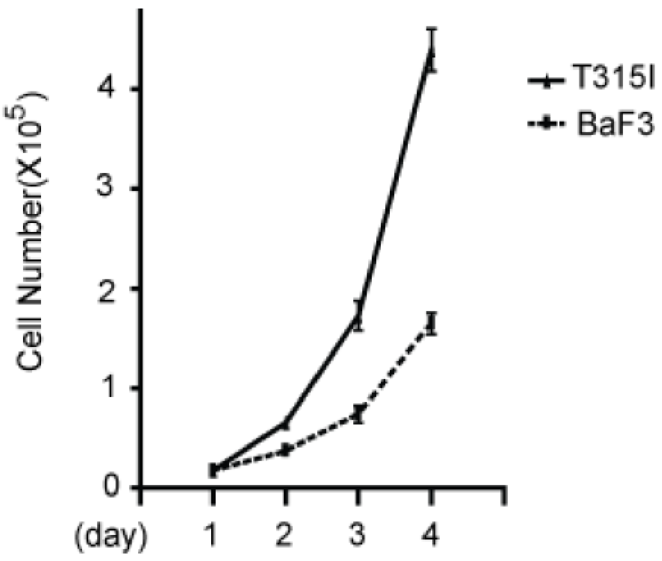

SFigure 8. T315I cells showed increased proliferation rates compared to parental cells. T315I and Parental Ba/F3 cells were seed in 10mm^3 plates. Culture conditions were described in the Methods. Cells were collected and counted at indicated times to determine cell numbers and proliferation rates. Each data point represent mean ± s.d. from two independent experiments.

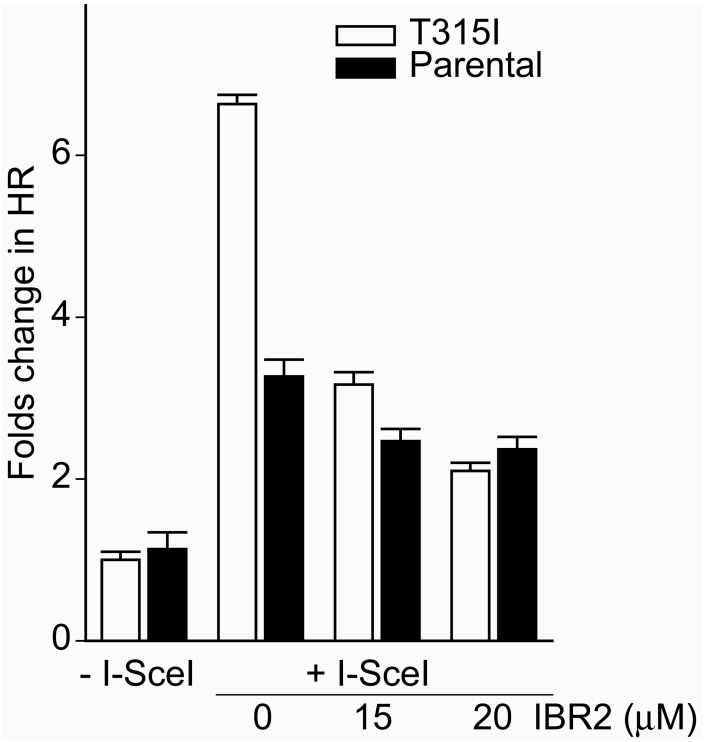

SFigure 9. IBR2 dosage-dependently inhibits HR in T315I but not parental cells. T315I or parental cells were transient-transfected with I-SceI expression vector pCABSce for 4 hours, followed by treatment with IBR2 at indicated concentrations. Fold-changes in HR were calculated as the ratio of the percentage of GFP-positive cells with I-SceI transfection over that with mock transfection. A total of 50,000 events are analyzed for each experiment; and experiments are performed in triplicates. Bars represent means ± s.d.

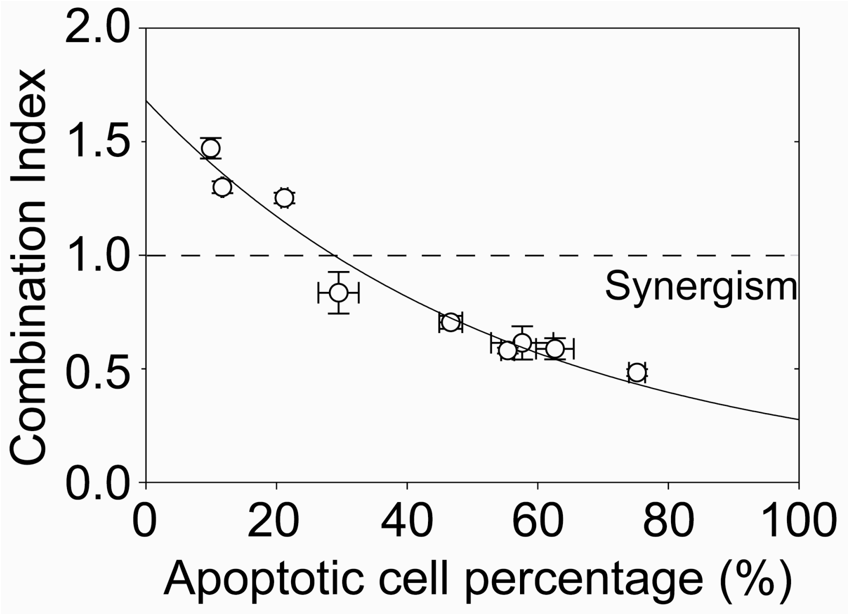

SFigure 10. Synergistic effect of IBR2 and Imatinib in killing K562 cells. Dose-response studies for each single agent and their combinations were performed according to the Chou-Talalay method (Chou, 2006). K562 cells were treated with (i) imatinib (0, 0.1, 0.2, 0.4, 0.6 μM) with IBR2 (0 or 15 μM) for 48 hours, or (ii) imatinib (0 or 0.2 μM) with IBR2 (0, 5, 10, 15, 20 μM) for 48 hours, then stained with annexin V-FITC and PI. Percentages of apoptotic cells were measured by flow cytometric analysis. Results are average of 2 independent experiments. Combination index (CI) was calculated following Chou's algorithm and plotted against the percentage of apoptotic cells. CI < 1 indicates synergistic effect.

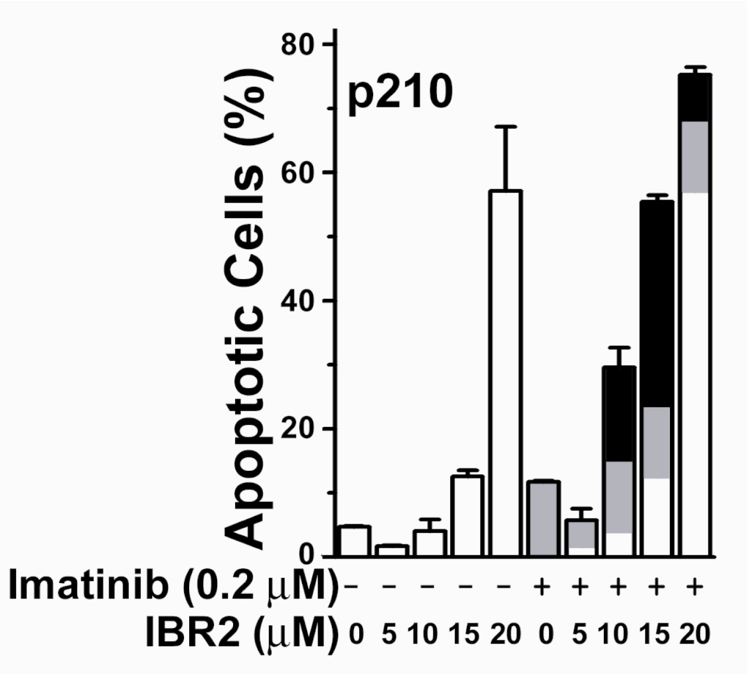

SFigure 11. IBR2 and Imatinib synergistically induce apoptosis in P210 cells. Wild-type BCR-ABL-expressing Ba/F3 cells (P210) were treated, stained, and analyzed according to the methods described in the methods. Bars represent means ± s.d. from three independent experiments. White color indicates the fraction that was induced by IBR2 alone. Grey shade indicates the fraction that was induced by imatinib alone. Black bars indicate the fraction of synergistic effect of both agents.

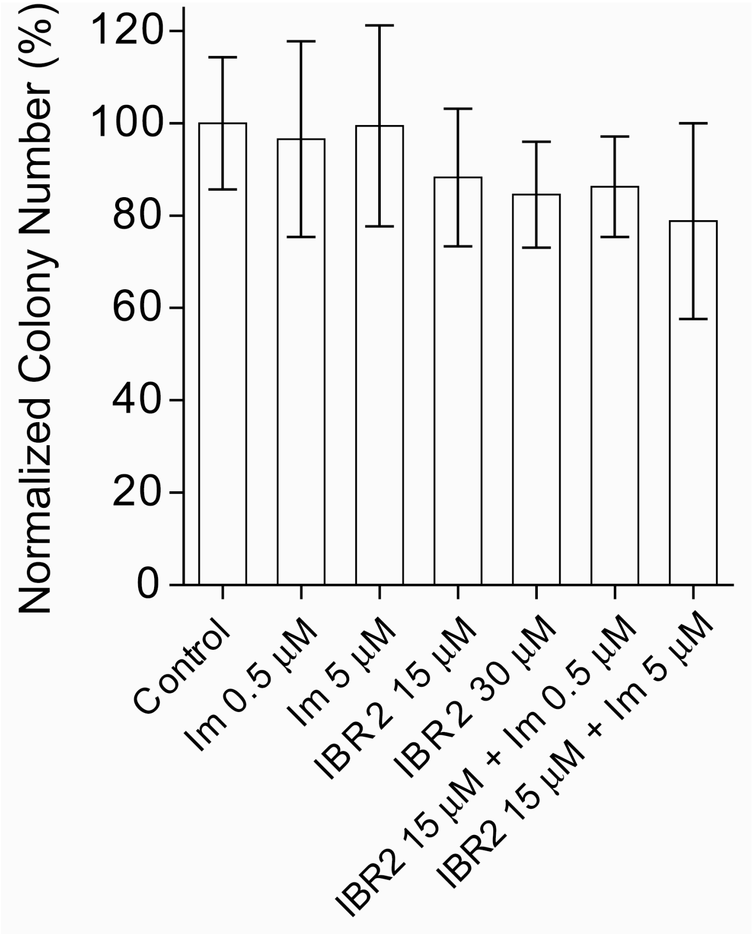

SFigure 12. IBR2 and Imatinib do not inhibit the growth of CD34$^+$ normal bone marrow cells. 3000 CD34$^+$ normal bone marrow cells (Stemcell Technologies Inc.) are treated with Im or IBR2 or their combination at indicated concentrations for 96 hours. Cells are then plated in semisolid methylcellulose progenitor culture medium for 10-14 days. Experiments are performed in triplicate, granulocyte-macrophage colony forming units are quantitated. Normalized colony numbers (%) are presented as means ± s.e.m. No significant inhibitory effect of IBR2 and imatinib were observed.

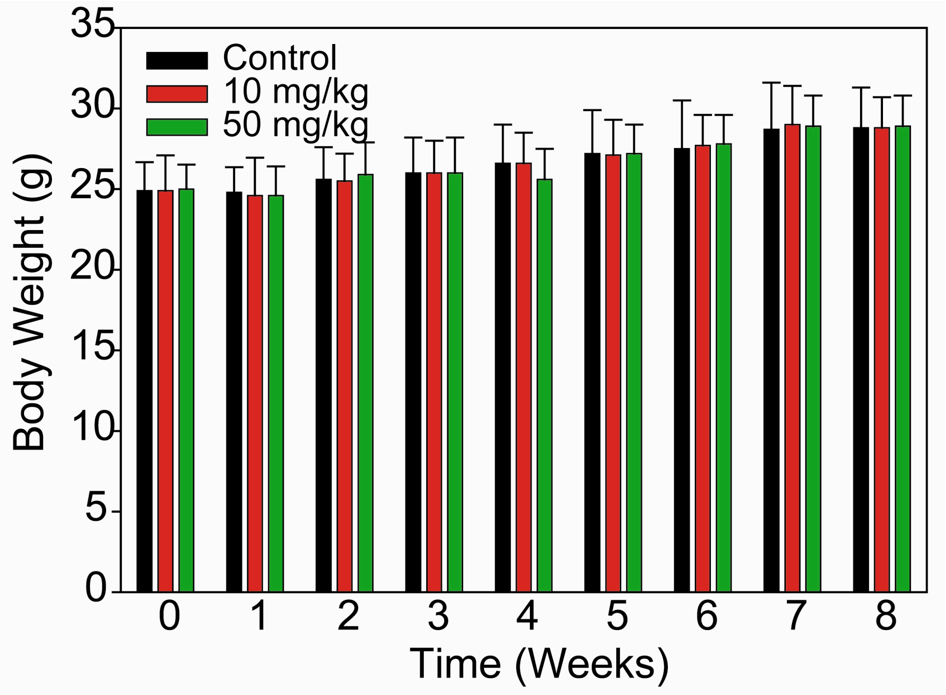

SFigure 13. Body weight of nude mice is not affected by IBR2 treatment. Nude mice (9-11 week old athymic female BALB/c-nude mice (nu/nu), Charles River Laboratories) were randomized into three groups (n = 8 per group) to receive intraperitoneal injection of vehicle alone (15% DMSO, 20% Tween 20, 10% PEG 400, 55% saline) or IBR2 (10 or 50 mg/kg in vehicle) every day for 8 weeks. Body weight of control group had no significant difference from that of both IBR2 treated groups. Data were presented as means ± s.d.

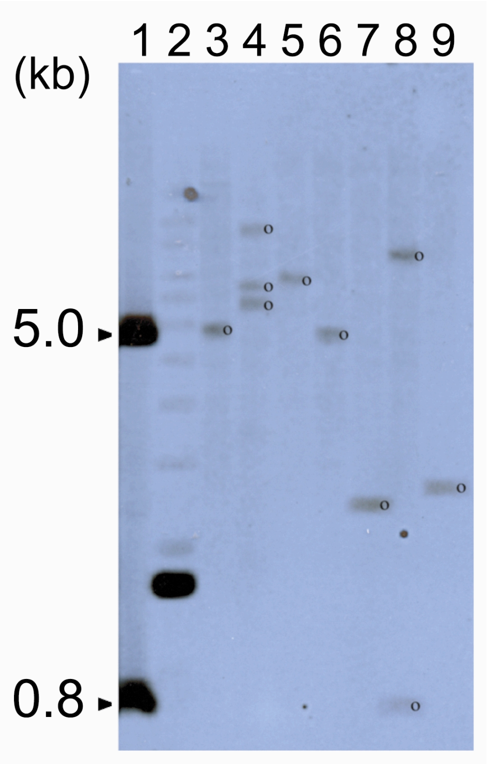

SFigure 14. Screening for cells with a single-copy DR-GFP HR reporter. Representative data for screening of cells with a single-copy DR-GFP HR reporter by genomic southern blotting analysis. Lane 1: Plasmid pDR-GFP is digested with Hind III, as a positive control; Lane 2: Marker; Lane 3-9: several screened clones; Lane 8: clone #14 contains a single-copy of DR-GFP reporter. HindIII digestion of pDR-GFP (8.6 kb) yielded 3 fragments: 0.8 kb, 2.8kb, and 5kb. The single integration in the cell clone results in one 0.8kb and one > 5kb positive fragments by probing with the 0.8kb probe prepared from pDR-GFP/HindIII.

Time (hour)	Percentage of each cell cycle phase (%)											
	Control				20 μM IBR2				40 μM B6			
	Sub-G1	G1	S	G2/M	Sub-G1	G1	S	G2/M	Sub-G1	G1	S	G2/M
6	0.4	58.4	20.1	21.1	0.2	62.4	18.7	18.7	0.6	57.6	21.4	20.4
12	0.5	58.7	18.5	22.3	0.4	68.6	12.2	18.8	0.5	57.7	19.7	22.1
24	0.5	58.7	18.2	22.6	0.2	85.3	5.0	9.5	0.4	58.9	20.1	20.6
36	0.6	61.4	17.5	20.5	0.3	91.3	4.4	4.0	0.6	60.4	17.4	21.6
48	0.6	64.5	15.7	19.2	23.4	69.6	4.0	3.0	0.5	64.5	15.9	19.1
72	0.4	64.8	16.6	18.2	30.0	65.9	1.5	2.6	0.5	65.2	16.5	17.8

STable 1. Cell cycle progression of IBR2 treatment in MCF7 cells. 5×10^5 MCF-7 cells were seeded in 10-cm dishes for 24 hours and treated with 20 μM IBR2 for various times. Cells were then trypsinized and fixed with 70% ethanol (-20 ºC) and stained for 30 minutes with propidium iodide (PI) staining solution (50 μg/ml PI, 0.1% sodium citrate, 50 μg/ml RNase A, 0.03% NP-40 in PBS). Flow cytometry analysis was performed using a FACScalibur flow cytometer and cell cycle distribution was analyzed with CellQuest software (Beckton Dickison). 10,000 events were analyzed for each sample and the experiment was repeated twice.

Supplementary Methods:

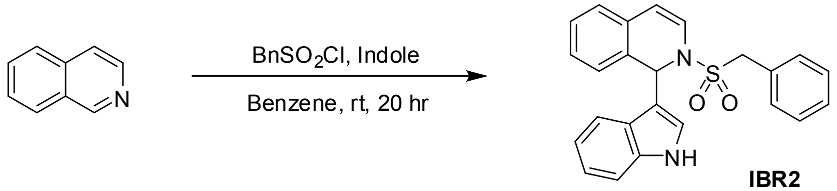

IBR2

Synthesis of 2-(benzylsulfonyl)-1-(1H-indol-3-yl)-1,2-dihydroisoquinoline (IBR2):

Isoquinoline (10.3 g, 80 mmol), indole (7.64 g, 40 mmol), and benzylsulphonyl chloride (4.68 g, 40 mmol) were dissolved in 140 ml anhydrous benzene, and stirred at r.t. for 20 hrs. A yellow precipitate gradually formed over time. The mixture was then filtered and washed with toluene and hexane. The resulting solid was dissolved in a minimum portion of DCM, and passed through a short silica gel column, and eluted with DCM. The fractions were combined and concentrated to give a beige solid as crude IBR2 (11.1g, 69.4%). A portion of the crude product (6.00 g) was dissolved in 210ml hot EtOH:DCM (20:1), and recrystalized to afford IBR2 as a white crystal (5.97 g). ^{1}H NMR (400 MHz, DMSO-d_6): δ (ppm) 11.01 (s, br, 1H), 7.75 (d, J = 8.0 Hz, 1H), 7.32 (d, J = 8.0 Hz, 1H), 7.25-6.92 (m, 11H), 6.84 (d, J = 2.6 Hz, 1H), 6.39 (d, J = 7.8 Hz, 1H), 6.38 (s, 1H), 6.13 (d, J = 7.8 Hz, 1H), 4.28 (d, J = 13.8 Hz, 1H), 4.24 (d, J = 13.8 Hz, 1H); ^{13}C NMR (100 MHz, DMSO-d_6): δ (ppm) 136.9, 132.1, 131.3, 130.1, 129.0, 128.9, 128.7, 128.2, 128.0, 127.2, 125.7, 125.4, 125.3, 122.8, 122.0, 120.1, 119.7, 116.4, 112.3, 111.0, 58.7, 54.2; MS (ESI): m/z 423 (M + Na^+); ESI-HRMS (m/z): Calcd. for $C_{24}H_{20}N_2O_2SNa$: 423.1143, Found: 423.1135.

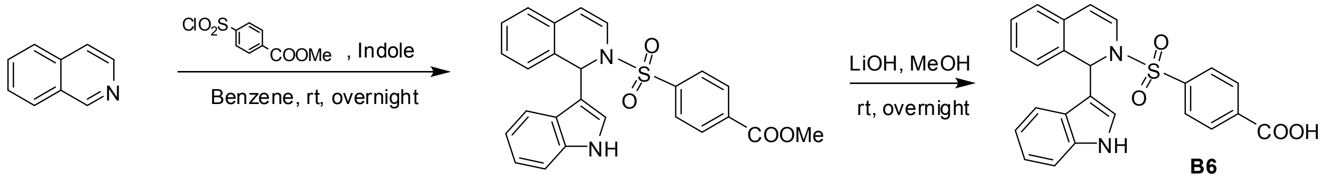

B6

Synthesis of 2-(4-carboxylphenylsulfonyl)-1-(1H-indol-3-yl)-1,2-dihydroisoquinoline (B6):

Isoquinoline (260 mg, 2 mmol), indole (117 mg, 1 mmol), and methyl 4-(chlorosulfonyl)benzoic ester (235 mg, 1 mmol) were dissolved in 5 ml anhydrous benzene, and stirred at r.t. overnight. Water was added and the mixture was extracted with DCM (2 x 20ml). The combined organic phase was washed with 1N HCl (20 ml), 1N $NaHCO_3$ (20 ml), and brine (20ml) successively, and then dried over anhydrous Na_2SO_4. After filtration and removal of the solvent, the residue was purified by silica gel flash chromatography (hexane / ethyl acetate = 85:15) to afford the B6-precursor 2-(4-methylcarboxylphenylsulfonyl)-1-(1H-indol-3-yl)-1,2-dihydroisoquinoline as a light yellow solid (317 mg, 71.4 %).

B6-precursor (113.5 mg, 0.256 mmol) was dissolved in MeOH (8 ml), and $LiOH.H_2O$ (18 mg, 0.43 mmol) was added to the solution. The mixture was stirred at r.t. overnight. The reaction was quenched and pH was adjusted to 1 using 1N HCl. The solution was then extracted with EtOAc (2 x 15 ml), and concentrated. The residue was purified by silical gel flash chromatography (ethyl acetate) to afford B6 as a light yellow solid (74.3 mg, 67.5 %). ^{1}H NMR (400 MHz, DMSO-d_6): δ (ppm) 13.32 (s, br, 1H), 10.87 (s, br, 1H), 7.77 (d, J = 8.0 Hz, 1H), 7.74 (d, J = 7.7 Hz, 2H), 7.71 (d, J = 7.7 Hz, 2H), 7.23 (d, J = 8.1 Hz, 1H), 7.18-7.15 (m, 1H), 7.11-7.03 (m, 3H), 7.00 (dd, J = 7.5, 7.5 Hz, 1H), 6.94 (dd, J = 7.5, 7.5 Hz, 1H), 6.66 (d, J = 8.7 Hz, 2H), 6.60 (d, J = 2.4 Hz, 1H), 6.28 (d, J = 7.4 Hz, 1H); ^{13}C NMR (100 MHz, DMSO-d_6): δ (ppm) 166.5, 142.6, 137.0, 134.9, 131.5, 130.0, 129.3, 128.3, 128.2, 127.3, 127.2, 125.5, 125.3, 125.2, 124.9, 122.3, 121.9, 120.0, 119.5, 115.6, 112.2, 54.5; MS (ESI): m/z 453 (M + Na^+); ESI-HRMS (m/z): Calcd. for $C_{24}H_{18}N_2O_4SNa$: 453.0885, Found: 453.0880.

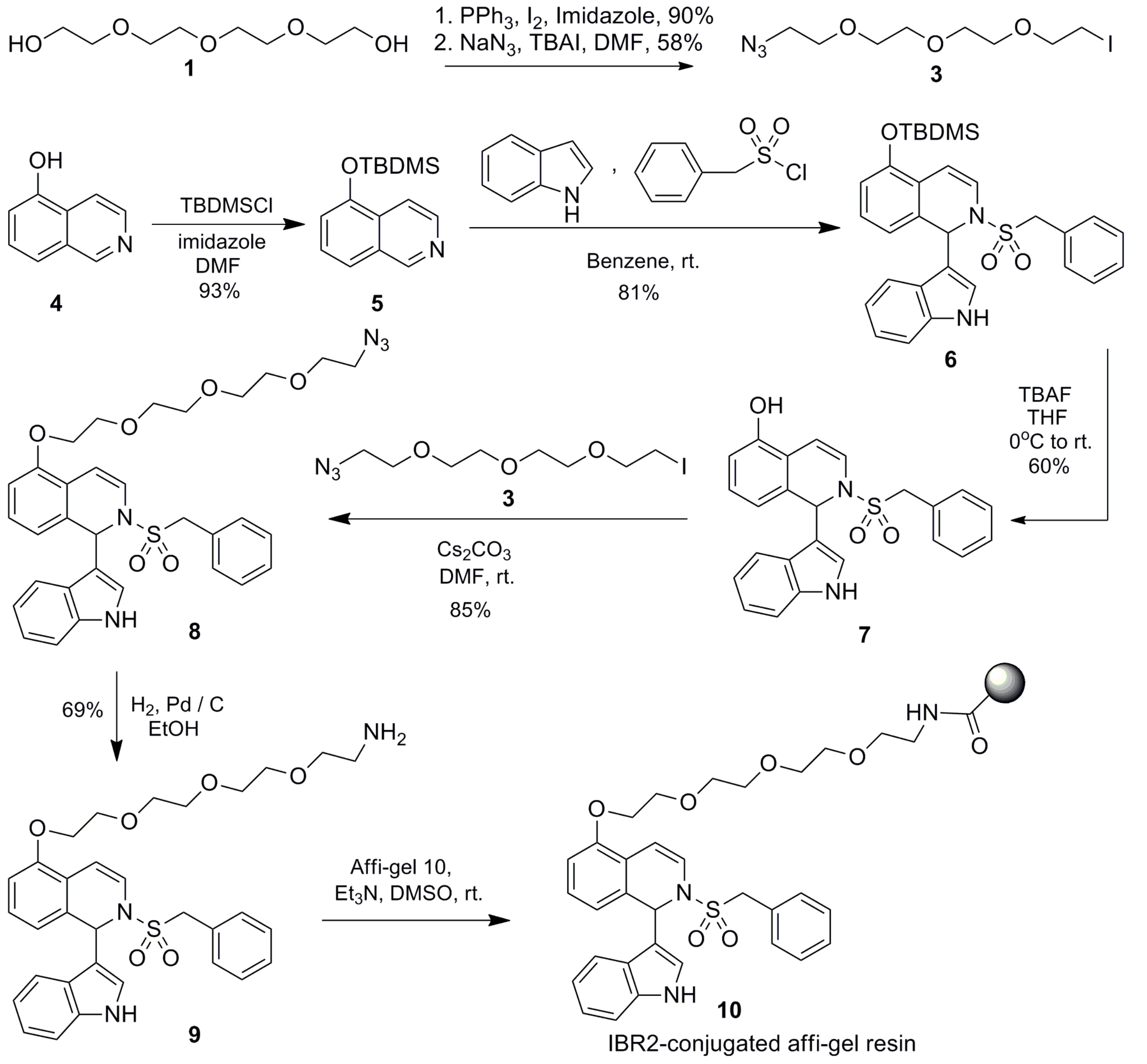

Synthesis of IBR2- conjugated affi-gel resin

A solution of tetraethylenediol **1** (8.2 g, 42.3 mmol) in Et_2O (100 mL) and MeCN (60 mL) was cooled to 0°C. Then, PPh_3 (28.6 g, 109.2 mmol) and imidazole (7.8 g, 114.7 mmol) were added slowly. After the mixture was stirred at 0°C for 2min, I_2 (30.4 g, 119.8 mmol) was added. The resultant brown-black slurry was stirred for 3h at 0°C. The mixture was diluted with Et_2O (900 mL). The resultant mixture was filtered and the organic phase was washed with sat. aq. $Na_2S_2O_3$ (3× 250 mL), sat. aq. Cu_2SO_4 (3× 250 mL) and H_2O (3 × 250 mL). The organic phase was dried over anhydrous Na_2SO_4. After filtration and removal of the solvent, the white residue

was dissolved in Et_2O (100ml). The resulting suspension was filtered and the filtrate was concentrated to give another white oily solid. This procedure was repeated with Et_2O (50 mL) to give a clear yellowish oil. Then, hexane (100 mL) was added and the resultant mixture was filtered. The filtrate was concentrated to give a residue, which was purified by silica gel chromatography (hexane / ethyl acetate = 3 : 1) to afford 1, 11-diiodo-3,6,9-trioxaundecane **2** as a clear yellowish oil (15.785 g, 90%). ^{1}H NMR (400 MHz, $CDCl_3$) δ 3.69 (dd, J = 0.8, 2.0 Hz, 4H), 3.61-3.59 (m, 8H), 3.22-3.19 (m, 4H); ^{13}C NMR (100 MHz, $CDCl_3$) δ 71.8, 70.6, 70.1, 3.3; MS (ESI) m/z 432 (M + NH_4^+), 437 (M + Na^+); ESI-HRMS Calcd for C_8H_{16}I2O3Na (M + Na^+), 436.9086 Found: 436.9068.

To a solution of compound **2** (2.93 g, 7.09 mmol) and TBAI (130 mg, 0.35 mmol) in DMF (50 mL) was added NaN_3 (460 mg, 7.08 mmol). The mixture was stirred overnight at room temperature. After that, H_2O (50mL) was added to quench the reaction. The mixture was extracted with CH_2Cl_2 (3 × 40 mL). The combined organic phases were dried over anhydrous Na_2SO_4. After filtration and removal of all the solvent, the residue was purified by silica gel chromatography (hexane / ethyl acetate = 6 : 1) to give the 11-azido-1-iodo-3,6,9-trioxaundecane **3** (800 mg, 58% based on consumed **2**) as a yellow oil. In addition, starting material **2** (1.21 g) was recovered. ^{1}H NMR (400 MHz, $CDCl_3$) δ 3.74 (dd, *J* = 6.8, 2.8 Hz, 2H), 3.69-3.65 (m, 10H), 3.39-3.36 (m, 2H), 3.25 (dd, *J* = 6.8, 2.8, 2H); ^{13}C NMR (100 MHz, $CDCl_3$) δ 72.1, 70.8, 70.8, 70.4, 70.2, 50.8, 3.1; MS (ESI) m/z 330 (M + H^+), 347 (M + NH_4^+), 352 (M + Na^+);; ESI-HRMS Calcd for $C_8H_{20}N_4IO_3$ (M + NH_4^+), 347.0580 Found: 347.0576.

To a suspension of 5-hydroxyl-isoquinoline **4** (1.0 g, 90% tec., 6.2 mmol) and imidazole (1.26 g, 18.6 mmol) in DMF (20 ml) was added TBDMSCl (1.40 g, 9.29 mmol). The resultant mixture was stirred overnight at room temperature. After that, the mixture was poured into H_2O (200 mL) and the aqueous phase was extracted with Et_2O (3× 50 mL). The combined organic phases were dried over anhydrous Na_2SO_4. After filtration and removal of all the solvent in *vacuo*, the residue was purified by silica gel chromatography (hexane / ethyl acetate = 3 : 1) to

give 5-(*tert*-butyldimethylsilanyloxy)-isoquinoline **5** (1.50 g, 93%) as a clear oil. MS (ESI) m/z 260 (M + H^+); ESI-HRMS Calcd for $C_{15}H_{22}NOSi$ (M + H^+), 260.1471 Found: 260.1471.

To a solution of compound **5** (1.0 g, 3.85 mmol) in benzene (6.7 mL) was added $BnSO_2Cl$ (370 mg, 1.94 mmol). The mixture was stirred at room temperature for 30min. After that, indole (255 mg, 1.92 mmol) was added and the resultant mixture was stirred overnight at room temperature. Then, the mixture was directly subjected to purification of silica gel chromatography (hexane / ethyl acetate = 5 : 1 to 4 : 1) to afford 5-(*tert*-butyldimethylsilanyloxy)-2- benzylsulfonyl-1-indol-3-yl-1, 2-dihydro-isoquinoline **6** (835 mg, 81.2% based on the $BnSO_2Cl$) as a white foam. MS (ESI) m/z 548 (M + NH_4^+); 553 (M + Na^+); ESI-HRMS Calcd for $C_{30}H_{34}N_2O_3SSiNa$ (M + Na^+), 553.1957 Found: 553.1948.

To a solution of compound **6** (266 mg, 0.49 mmol) in THF (6.0 mL) was added TBAF (0.54 mL, 1M in THF, 0.54 mmol). The mixture was stirred at room temperature for 5min. Then, H_2O (50 mL) was added to quench the reaction and the aqueous layer was extracted with CH_2Cl_2 (3 × 30 mL). The combined organic phases were dried over anhydrous Na_2SO_4. Filtration and removal of all the solvent resulted in a residue, which was purified by silica gel chromatography to provide 5-hydroxy-2-benzylsulfonyl-1-indol-3-yl-1, 2-dihydro-isoquinoline **7** (175 mg, 86%) as a yellowish foam. MS (ESI) m/z 434 (M + NH_4^+); 439 (M + Na^+); ESI-HRMS Calcd for $C_{24}H_{24}N_3O_3S$ (M + NH_4^+), 434.1538 Found: 434.1538.

To a solution of compound **7** (100 mg, 0.255 mmol) in DMF (2 mL) was added Cs_2CO_3 (92 mg, 0.28 mmol) and compound **3** (100 mg, 0.30 mmol). The resultant mixture was stirred overnight at room temperature. After that, the mixture was directly subjected to purification of silica gel chromatography to afford 5-[1-(11-azido-3, 6, 9-trioxaundecanoxyl)]-2-benzylsulfonyl-1-indol-3-yl-1, 2-dihydro-isoquinoline **8** (133 mg, 85%) as a yellow oil.

To a solution of compound **8** (62 mg, 0.10mmol) in EtOH (4 mL) was added Pd / C (10% Pd, 40 mg). The mixture was hydrogenated for 20min at room temperature under 1atm H_2 pressure. After that, removing all the solvent gave a residue, which was purified by silica gel

chromatography (CH_2Cl_2 / MeOH / ammonium hydroxide = 100:10:3) to afford 5-[1-(11-amino-3,6,9-trioxaundecanoxyl)]-2-benzylsulfonyl-1-indol-3-yl-1,2-dihydro- isoquinoline **9** (41 mg, 69%) as a light yellow foam. ^{1}H NMR (500 MHz, CD_2Cl_2) δ 9.14 (s, 1H), 7.96 (dd, *J* = 1.0, 2.0 Hz, 1H), 7.33 (dd, *J* = 1.0, 2.0 Hz, 1H), 7.26-7.23 (m, 1H), 7.17-7.09 (m, 5H), 6.95 (d, *J* = 7.0 Hz, 2H), 6.83 (d, *J* = 8.0 Hz, 1H), 6.70 (s, 1H), 6.51 (dd, *J* = 8.0, 7.5 Hz, 2H), 6.32 (s, 1H), 6.27 (dd, *J* = 1.5, 1.0 Hz, 1H), 4.24-4.16 (m, 2H), 4.12 (d, *J* = 13.5 Hz, 1H), 4.01 (d, *J* = 14.0 Hz, 1H), 3.90-3.87 (m, 2H), 3.72-3.71 (m, 2H), 3.65-3.60 (m, 4H), 3.57-3.55 (m, 2H), 3.44 (t, *J* = 5.0 Hz, 2H), 2.80 (t, *J* = 5.0 Hz, 2H), 1.95 (br, 2H); ^{13}C NMR (125 MHz, CD_2Cl_2) δ 153.8, 136.8, 133.4, 131.1, 129.0, 128.9, 128.8, 128.7, 125.9, 125.6, 124.2, 122.6, 120.5, 120.3, 119.8, 119.6, 116.2, 111.8, 111.4, 107.5, 73.5, 71.4, 71.1, 71.1, 70.8, 70.3, 68.8, 59.4, 54.4, 42.1; MS (ESI) m/z 592 ($M + H^+$); ESI-HRMS Calcd for $C_{32}H_{38}N_3O_6S$ ($M + H^+$), 592.2481 Found: 592.2478.

For **IBR2-conjugated affi-gel resin:** The wet affi-gel beads (Purchased from Bio-Rad, Catalogue No. 153-6099, 10 mL) was added to a 10cm-long column and the *iso*-propanol buffer was let to drain out (about 2mL dry beads was obtained, 0.03mmol). The dry beads were washed using DMSO (3 × 3 mL). Then, DMSO (3mL) was added followed by a solution of compound **9** (6.0 mg, 0.01 mmol) in DMSO (0.81 mL) and Et_3N (14 μL). The whole column was shaken at room temperature for about 4h until TLC shown that compound **9** was completely consumed. After that, ethanolamine (10 μL) was added and the column was shaken overnight at room temperature. Then, all the solvent in column was let to drain out and the beads were washed with DMSO (2 mL) and 1 × PBS (2 × 3 mL). The resulting IBR2-conjugated affi-gel resin **10** was stored in 1 × PBS at 4°C for use.

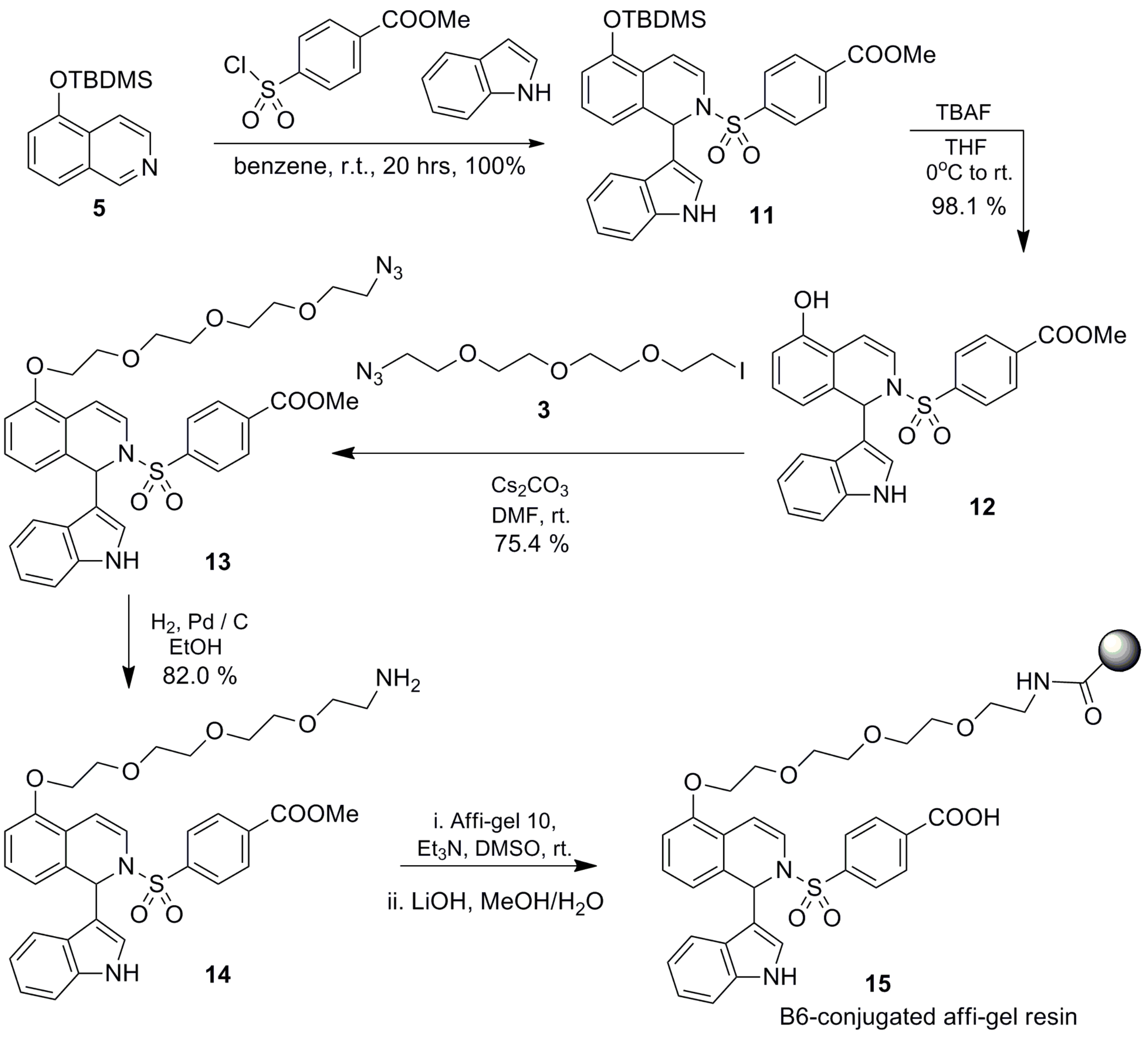

Synthesis of B6- conjugated affi-gel resin

To a solution of compound **5** (1.0 g, 3.85 mmol) in benzene (6.7 ml) was added methyl 4-(chlorosulfonyl)benzoate (455 mg, 1.94 mmol). The mixture was stirred at room temperature for 30min. After that, indole (255 mg, 1.92 mmol) was added and the resultant mixture was stirred overnight at room temperature. Then, the mixture was directly subjected to purification of silica gel chromatography (hexane / ethyl acetate = 4 : 1) to afford methyl 4-((5-((tert-butyldimethyl silyl)oxy)-1-(1H-indol-3-yl)isoquinolin-2(1H)-yl)sulfonyl)benzoate **11** (1.11 g, 100 % based on sulphonyl chloride) as a light yellow foam.

To a solution of compound **11** (1.1 g, 2.0 mmol) in THF (24.0 ml) was added TBAF (2.2 ml,

1M in THF, 2.2 mmol). The mixture was stirred at room temperature for 5min. Then, H_2O (200 ml) was added to quench the reaction and the aqueous layer was extracted with CH_2Cl_2 (3 × 120 ml). The combined organic phases were dried over anhydrous Na_2SO_4. Filtration and removal of all the solvent resulted in a residue, which was purified by silica gel chromatography (Hexanes:EtOAc=5:2) to provide methyl 4-((5-hydroxy-1-(1H-indol-3-yl)isoquinolin-2(1H)-yl)sulfonyl)benzoate **12** (902.2 mg, 98.1 %) as a yellowish foam.

To a solution of compound **12** (118 mg, 0.255 mmol) in DMF (2 ml) was added Cs_2CO_3 (92 mg, 0.28 mmol) and compound **3** (100 mg, 0.30 mmol). The resultant mixture was stirred overnight at room temperature. After that, the mixture was directly subjected to purification of silica gel chromatography (H/EA=1:1) to afford methyl 4-((5-(2-(2-(2-(2-azidoethoxy)ethoxy) ethoxy)ethoxy)-1-(1H-indol-3-yl) isoquinolin- 2(1H)-yl)sulfonyl)benzoate **13** (127.2 mg, 75.4 %) as a yellow oil. MS (ESI) m/z 679 (M + NH_4^+), 684 (M + Na^+); ESI-HRMS Calcd for $C_{33}H_{35}N_5O_8SNa$ (M + Na^+), 684.2104, Found: 684.2104.

To a solution of compound **13** (127 mg, 0.192 mmol) in 95% EtOH (8 ml) was added Pd / C (10% Pd, 80 mg). The mixture was hydrogenated for 20 min at room temperature under 1atm H_2 pressure. After that, removing all the solvent gave a residue, which was purified by silica gel chromatography (CH_2Cl_2 / MeOH / ammonium hydroxide = 100 : 10 : 3) to afford methyl 4-((5-(2-(2-(2-(2-aminoethoxy)ethoxy)ethoxy)ethoxy)-1-(1H-indol-3-yl)isoquinolin-2(1H)-yl)sul fonyl)benzoate **14** (100 mg, 82.0 %) as a light yellow foam. MS (ESI) m/z 636 (M + H^+); ESI-HRMS Calcd for $C_{33}H_{38}N_3O_8S$ (M + H^+), 636.2380, Found: 636.2371.

For **B6-conjugated affi-gel resin:** The wet affi-gel beads (Purchased from Bio-Rad, Catalogue No. 153-6099, 10 ml) was added to a 10 cm-long column and the *iso*-propanol buffer was let to drain out (about 2 ml dry beads was obtained, 0.03 mmol). The dry beads were washed using DMSO (3 × 3 ml). Then, DMSO (3 ml) was added followed by a solution of compound **14** (6.4 mg, 0.01 mmol) in DMSO (0.81 ml) and Et_3N (14 μl). The whole column was shaken at room temperature for about 4h until TLC shown that compound **14** was completely consumed.

After that, ethanolamine (10 μl) was added and the column was shaken overnight at room temperature. The solvent in column was let to drain, and the beads was washed in MeOH (2x 4 mL), and then resuspended in MeOH (4 ml), containing LiOH.H_2O (9 mg, 0.20 mmol), and was shaken at room temperature for 4 h, to remove methyl protective group. Then, all the solvent in column was let to drain out and the beads were washed with DMSO (2 ml), 95% EtOH (2 × 3 ml), and 1 × PBS (2 × 3 ml). The resulting B6-conjugated affi-gel resin **15** was stored in 1 × PBS at 4°C for use.

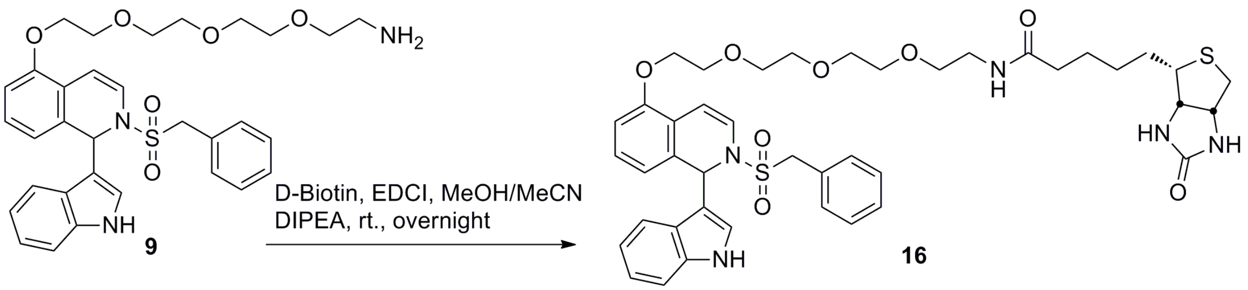

Synthesis of biotin-conjugated IBR2

The solution of compound **9** (20 mg, 0.034 mmol), biotin (9 mg, 0.037 mmol), EDC·HCl (13 mg, 0.043 mmol) and DIPEA (8.0 μl, 0.045 mmol) in MeCN (0.24 ml) and MeOH (0.08 ml) was stirred overnight at room temperature. Then, all the solvent was removed in vacuo and the residue was purified by silica gel chromatography (CH_2Cl_2/MeOH = 20 : 1) to afford N-(2-(2-(2-(2-(2-(benzylsulfonyl)-1-(1H-indol-3-yl)-1,2-dihydroisoquinolin-5-yloxy)ethoxy)ethoxy)ethoxy)ethyl)-5-((3aS,4S,6aR)-2-oxohexahydro-1H-thieno[3,4-d]imidazol-4-yl)pentanamide **16** (22 mg, 79%) as a foam.

^{1}H NMR (400 MHz, $CDCl_3$) δ 9.06 (d, J = 30.8 Hz, 1H), 8.01-7.99 (m, 1H), 7.33-7.31 (m, 1H), 7.21 (t, J = 7.4 Hz, 1H), 7.17-7.04 (m, 5H), 6.90 (d, J = 7.6 Hz, 2H), 6.76 (dd, J = 8.4 Hz, 1.6 Hz, 1H), 6.67 (s, 1H), 6.63-6.59 (m, 1H), 6.50 (d, J = 7.6 Hz, 1H), 6.45 (d, J = 7.6 Hz, 1H), 6.37 (s, 1H), 6.34 (s, 1H), 6.23 (d, J = 7.6 Hz, 1H), 5.39 (s, 1H), 4.36-4.32 (m, 1H), 4.22-4.18 (m, 1H), 4.16-4.07 (m, 3H), 3.97 (d, J = 14.0 Hz, 1H), 3.91-3.86 (m, 2H), 3.76-3.74 (m, 2H), 3.69-3.54 (m, 7H), 3.54-3.51 (m, 2H), 3.39-3.45 (m, 2H), 2.99-2.92 (m, 1H), 2.77 (dt, J = 12.8, 4.0 Hz, 1H), 2.62 (d, J = 12.8 Hz, 1H), 2.10 (t, J = 7.6 Hz, 2H), 1.64-1.52 (m, 4H), 1.34-1.27 (m, 2H);

^{13}C NMR (125 MHz, $CDCl_3$) δ 173.6, 164.2, 153.3 and 153.3, 136.4, 133.2 and 133.2, 130.8, 128.7, 128.6, 128.5, 128.1, 125.6, 125.4 and 125.3, 124.0, 122.4 and 122.3, 120.3, 120.0 and 120.0, 119.6, 119.5 and 119.5, 115.8, 111.7, 110.9 and 110.9, 106.9, 71.1, 70.8, 70.7 and 70.7, 70.3, 70.1 and 70.1, 70.0, 68.4 and 68.4, 61.9 and 61.8, 60.3, 59.3, 55.6, 54.0, 40.7, 39.4, 36.0 and 36.0, 28.2 and 28.1, 25.7 and 25.7; (1:1 mixture of diasteromers).

MS (ESI) *m/z* 840.3 (M + Na^+);

ESI-HRMS Calcd for $C_{42}H_{51}N_5O_8S_2Na$ (M + Na^+), 840.3077 Found: 840.3062.

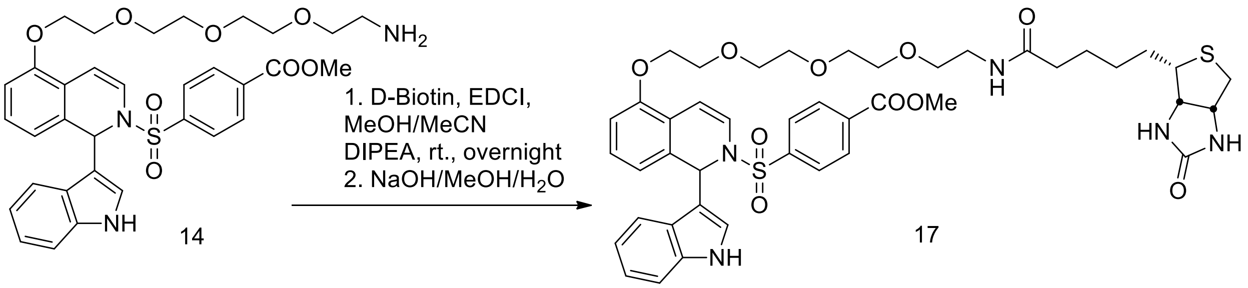

Synthesis of biotin-conjugated B6

The solution of compound **14** (9.0 mg, 0.015 mmol), biotin (4 mg, 0.016 mmol), EDC·HCl (6 mg, 0.018 mmol) and DIPEA (3.4 μl, 0.019 mmol) in MeCN (0.10 ml) and MeOH (0.03 ml) was stirred overnight at room temperature. Then the reaction was purified by silica gel chromatography (CH_2Cl_2/MeOH = 20 : 1) to afford the biotinylated methyl ester as a light yellow foam upon vacuum, which was used directly after concentration. MS (ESI) *m/z* 879.4 (M + NH_4^+), 884.3 (M + Na^+); ESI-HRMS Calcd for $C_{43}H_{51}N_5O_{10}S_2Na$ (M + Na^+), 884.2975, Found: 884.2974.

The biotinylated methyl ester was dissolved in MeOH (0.1 ml), and NaOH solution (0.1 mmol, 0.05 mL of 2 M NaOH in water) was added to the solution. The mixture was stirred at r.t. 4h. The reaction was quenched with 2N HCl (~ 0.05 mL). Then the reaction was purified by silica gel chromatography (CH_2Cl_2/MeOH = 10 : 1) to afford 4-((1-(1H-indol-3-yl)-5-((13-oxo-17-((3aS,4S,6aR)-2-oxohexahydro-1H-thieno[3,4-d]imidazol-4-yl)-3,6,9-trioxa-12-aza heptadecyl)oxy)isoquinolin-2(1H)-yl)sulfonyl)benzoic acid **17** (5.0 mg, 39.3 % yield for two steps) as a white powder. MS (ESI) *m/z* 865.3 (M + NH_4^+), 870.3 (M + Na^+); ESI-HRMS Calcd for $C_{42}H_{49}N_5O_{10}S_2Na$ (M + Na^+), 870.2819, Found: 870.2805.

Reference:

Chou T-C (2006) Theoretical Basis, Experimental Design, and Computerized Simulation of Synergism and Antagonism in Drug Combination Studies. Pharmacol Rev 58**:** 621-681

Wu G, Qiu X-L, Zhou L, Zhu J, Chamberlin R, Lau J, Chen P-L, Lee W-H (2008) Small Molecule Targeting the Hec1/Nek2 Mitotic Pathway Suppresses Tumor Cell Growth in Culture and in Animal. Cancer Res 68**:** 8393-8399
